# Differential methylation analysis of floral buds between two morphs unravels the contributions of key genes to flowering time in heterodichogamous *Cyclocarya paliurus*

**DOI:** 10.1093/hr/uhaf296

**Published:** 2025-11-03

**Authors:** Qian Wang, Yanhao Yu, Di Mei, Yibo Yang, Yanmeng Huang, Xia Mao, Xulan Shang, Yinquan Qu, Xiangxiang Fu

**Affiliations:** State Key Laboratory for the Development and Utilization of Forest Food Resources, Nanjing Forestry University, Nanjing 210037, China; Co-Innovation Center for Sustainable Forestry in Southern China, College of Forestry and Grassland, Nanjing Forestry University, Nanjing 210037, China; State Key Laboratory for the Development and Utilization of Forest Food Resources, Nanjing Forestry University, Nanjing 210037, China; Co-Innovation Center for Sustainable Forestry in Southern China, College of Forestry and Grassland, Nanjing Forestry University, Nanjing 210037, China; State Key Laboratory for the Development and Utilization of Forest Food Resources, Nanjing Forestry University, Nanjing 210037, China; Co-Innovation Center for Sustainable Forestry in Southern China, College of Forestry and Grassland, Nanjing Forestry University, Nanjing 210037, China; State Key Laboratory for the Development and Utilization of Forest Food Resources, Nanjing Forestry University, Nanjing 210037, China; Co-Innovation Center for Sustainable Forestry in Southern China, College of Forestry and Grassland, Nanjing Forestry University, Nanjing 210037, China; Co-Innovation Center for Sustainable Forestry in Southern China, College of Forestry and Grassland, Nanjing Forestry University, Nanjing 210037, China; School of Landscape Architecture, Jiangsu Vocational College of Agriculture and Forestry, Zhenjiang 212400, China; State Key Laboratory for the Development and Utilization of Forest Food Resources, Nanjing Forestry University, Nanjing 210037, China; Co-Innovation Center for Sustainable Forestry in Southern China, College of Forestry and Grassland, Nanjing Forestry University, Nanjing 210037, China; Fishery College, Zhejiang Ocean University, Zhoushan, Zhejiang 316022, China; State Key Laboratory for the Development and Utilization of Forest Food Resources, Nanjing Forestry University, Nanjing 210037, China; Co-Innovation Center for Sustainable Forestry in Southern China, College of Forestry and Grassland, Nanjing Forestry University, Nanjing 210037, China

## Abstract

*Cyclocarya paliurus* is a medicinal plant traditionally used in China to treat hypertension and diabetes. It exhibits heterodichogamy, a dimorphic mating system with protogynous and protandrous morphs, which are based on the maturation sequence of female and male flowers within the same plant. DNA methylation, a crucial epigenetic modification in regulating plant flowering, is poorly characterized in heterodichogamous species. Here, whole-genome bisulfite sequencing and transcriptome analyses were performed on female and male flower buds from two morphs during inflorescence elongation in diploid *C. paliurus*. Single-base methylation maps revealed higher DNA methylation levels in early-flowering samples, particularly in CHH contexts, which may be dynamically regulated by the interplay between *CpDRM-D2* and *CpDME-D1*. Candidate genes involved in the photoperiod, gibberellin, and trehalose-6-phosphate signaling pathways were identified based on their transcriptional and methylation dynamics across floral buds. Heterologous overexpression of *CpHd16*, *CpTPPD*, and *CpFTIP3* in *Arabidopsis* delayed flowering. Furthermore, field application of the DNA methylation inhibitor 5-azacytidine to diploid *C. paliurus* delayed the flowering of both male and female flowers and altered methylation levels within the *CpTPPD* promoter and *CpFTIP3* gene body. These epigenetic changes, accompanied by downregulated *CpTPPD* and upregulated *CpFTIP3* expression, suggest that these genes may mediate *C. paliurus* flowering via methylation-dependent regulation. This study provides novel insights into the molecular regulatory mechanisms of heterodichogamous flowering and lays a theoretical foundation for epigenetic research in *C. paliurus*.

## Introduction

Epigenetics involves heritable modifications in gene expression that occur without alterations to the underlying DNA sequence [1–3]. DNA methylation is a highly conserved epigenetic modification that regulates gene expression, silences transposable elements (TEs), and maintains genome stability across diverse organisms [3, 4]. Plant DNA methylation is stably transmitted through meiosis, influencing epigenetic inheritance across generations [3]. The levels of DNA methylation are tightly regulated during growth, development, responses to environmental stimuli, and throughout a plant’s life cycle [1, 4, 5]. DNA methylation is dynamically regulated by DNA methyltransferases and demethylases, which together determine the methylation status of CG, CHG, and CHH sequence contexts (where H is any base other than G, [4]). CG and CHG methylation are generally maintained by the actions of methyltransferase 1 (MET1) and chromomethylase 3 (CMT3), while CHH methylation is maintained by chromomethylase 2 (CMT2) and domain rearranged methyltransferase 2 (DRM2) [1, 4, 6]. The regulation of specific methylation states involves a dynamic interplay between *de novo* methylation, maintenance methylation, and active DNA demethylation processes [4]. Across various plant species, previous research has shown the critical involvement of DNA methylation in modulating phenotypes and response to biotic or abiotic stress [1, 3, 5, 7, 8].

Wheel wingnut (*Cyclocarya paliurus*), commonly known as ‘*sweet tea*’, is a valuable medicinal plant native to China. Its leaves are rich in triterpenoids, flavonoids, polyphenols, and other bioactive compounds, which drive its wide range of documented pharmacological activities, including hypoglycemic, hypolipidemic, antioxidant, and anticancer properties [9]. This species exhibits heterodichogamy, a form of sexual dimorphism characterized by the temporal separation of male and female flower functions within an individual [10, 11]. Based on the temporal sequence of pistillate and staminate flower maturation, *C. paliurus* exhibits two morphs—protogyny and protandry—that facilitate cross-fertilization between different individuals and maintain genetic diversity within the population [12]. Heterodichogamy is well documented in Juglandaceae, Betulaceae, Lauraceae, Sapindaceae, and Zingiberaceae [11, 12]. However, low seed fullness is a common challenge in heterodichogamous plants. For instance, only 0.1% of flowers in *Persea americana* yield mature fruits [13], and the fruit set in *Juglans regia* increases with flowering synchrony [14]. *C. paliurus* also faces this issue with a seed fullness rate of <30% [12]. This mating system holds significant ecological importance for reproductive adaptation and genetic diversity maintenance in Juglandaceae, yet its molecular regulatory mechanism remains unexplored. Recently, several candidate genes potentially associated with heterodichogamy have been identified through genomic and resequencing approaches, such as *TPPD* [15–18]. Furthermore, gibberellins (GAs) have already been identified as the dominant hormones governing flower differentiation and development, with GA_3_ positively regulating the physiological differentiation and bud break stages of floral buds in *C. paliurus* [19]. Similarly, key genes within the GA pathway have been identified as crucial for floral development in *Carya illinoinensis*, and application of GA_3_ has led to a significant increase in the number of male flowers of *J. regia* [20, 21]. These findings suggest that the heterodichogamy in *C. paliurus* may be regulated by both hormonal and genetic mechanisms.

Flowering marks a critical transition in the life cycle of plants, signifying the shift to reproductive growth. This process is regulated by a complex interplay of endogenous and exogenous factors [1, 22]. Several key regulatory pathways have been elucidated in model plants, including photoperiodism, gibberellin, vernalization, autonomous, aging, and ambient temperature pathways [8, 23]. Recent studies have shown that DNA methylation has pleiotropic effects on flowering plants, including floral organ development, floral patterning, and flowering time regulation [8, 24–26]. For example, comparative analyses reveal ecotype-specific CHH methylation patterns in *Panicum hallii* inflorescences [27], while differential methylation profiles distinguish the floral morphologies of homostylous *Fagopyrum tataricum* and heterostylous *F. esculentum* [25]. These epigenetic variations modulate flowering-related gene expression, ultimately controlling floral morphogenesis and developmental timing. The methylation status of *MeGI* in polyploid persimmon determines floral sex, and demethylation via inhibitors can induce male-to-female sex reversal [28]. In *Arabidopsis thaliana*, genome-wide demethylation downregulated *FLC* gene expression to promote flowering [29], while the *ddm1* mutant enhanced DNA demethylation to facilitate photoperiodic flowering [24]. In *Dactylis glomerata*, hypermethylation upregulated vernalization-related genes to advance flowering [26], and demethylation of *COL2* induced photoperiodic flowering in cotton [30]. Extensive evidence suggests that methylation changes are associated with the initiation of flowering triggered by vernalization, photoperiod, or hormonal signals, albeit with notable species-specific differences [8, 26, 29–31]. Additionally, the application of exogenous methylation inhibitors or enhancers to modulate flowering time suggests that methylation regulatory mechanisms may evolve into a novel flowering pathway, either dependent on or independent of other flowering pathways [8, 26]. Currently, the regulatory network underlying the temporal separation of male and female functions in *C. paliurus* remains unclear. Therefore, based on the phenotypic differences between two morphs in *C. paliurus* (including diploids and autotetraploids), we hypothesize that DNA methylation may have potential regulatory functions. Diploid populations exhibit a 1- to 6-day separation of male and female functions, whereas autotetraploids may have overlapping male and female flowering periods within the same individual [12]. This phenotypic diversity introduces complexity to the study of *C. paliurus*, posing challenges for unraveling its flowering biology.

In this study, to avoid interference from gene dosage effects, we selected diploid *C. paliurus* to investigate the regulatory mechanisms of DNA methylation in heterodichogamy. We constructed single-base resolution DNA methylation maps for male and female flowers of two morphs at the stage of inflorescence elongation, revealing higher methylation levels in early-flowering samples. By integrating transcriptomic data, we aimed to elucidate: (i) the potential regulatory mechanisms of DNA methylation in the heterodichogamous flowering characteristics of *C. paliurus*; (ii) the connection between DNA methylation modification and gene expression; and (iii) how methyltransferases and demethylases regulate DNA methylation in *C. paliurus*. We further investigated the regulation of DNA methylation-dependent flowering genes using two independent strategies: heterologous overexpression in *Arabidopsis* and treatment with the methylation inhibitor 5-azacytidine (5-azaC) in field-grown diploid *C. paliurus*. This study provides insights into the epigenetic control of heterodichogamy and highlights the potential for targeting DNA methylation in plant breeding strategies.

## Results

### Genome-wide DNA methylation profiles of two morphs in *C. paliurus*

**Figure 1:**
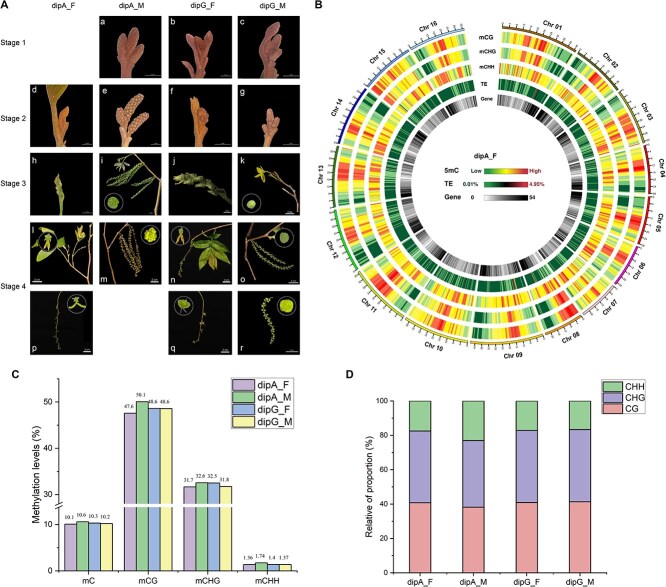
DNA methylation landscape in male and female flowers of *C. paliurus.* (A) Phenotypic progression of four floral buds across four developmental stages. Stage 1, dormant; Stage 2, bud break; Stage 3, inflorescence elongation; Stage 4, mature; dipA_F, protandrous female flowers; dipA_M, protandrous male flowers; dipG_F, protogynous female flowers; dipG_M, protogynous male flowers. (B) Chromosome density circle diagrams of CG/CHG/CHH sequence contexts, TEs, and gene density at Stage 3, using dipA_F as an example, with other samples detailed in Fig. S2. (C) DNA methylation levels in different floral buds. (D) Relative proportions of mCs in CG, CHG, and CHH sequence contexts.

*C. paliurus*, a heterodichogamous plant, exhibits two morphs: protandry (male flowers mature first) and protogyny (female flowers mature first). Phenological observations revealed significant phenotypic differences in same-sex flowers between the two morphs at the same stage (Fig. 1A). We constructed single-base methylation maps for dipA_F, dipA_M, dipG_F, and dipG_M. Each sample was sequenced with three replicates, and a total of 245.07 Gb raw bases were generated, with an average sequencing depth of >30× for each sample (Table S1). After quality control, 59.89–78.75 million 150-bp paired-end clean reads were mapped to the reference genome, achieving a mapping rate of >74%. The bisulfite conversion rate was >99.4%, indicating the data was sufficient for downstream analysis. Correlation analysis revealed strong correlations among biological replicates across the three sequence contexts, with coefficients exceeding 0.95 for CG and CHG contexts, and > 0.65 for CHH context (Fig. S1).

Genome-wide Circos analysis of *C. paliurus* revealed conserved DNA methylation patterns across the chromosomal landscape in all floral types, with TEs consistently representing <5% genomic coverage (Figs S2 and 1B). Low-density CG and CHG methylation regions corresponded to areas of high-density CHH methylation. The highest overall methylation level was observed in dipA_M, followed by dipG_F (Fig. 1C). The mean methylation levels across the CG, CHG, and CHH contexts were 47.59%–50.07%, 31.65%–32.54%, and 1.35%–1.73%, respectively (Fig. 1C). Among these three sequence contexts, the methylation level in dipA_M exceeded that of the other samples. Furthermore, we calculated the relative proportion of methylated cytosines (mC) across different sequences and found that mC contributed significantly to the CG sites (38.17%–41.34%) and CHG sites (38.8%–42.04%), while its contribution to the CHH sites was comparatively lower (16.61%–23.03%, Fig. 1D).

### Analysis of differentially methylated regions between different *C. paliurus* flowers

**Figure 2:**
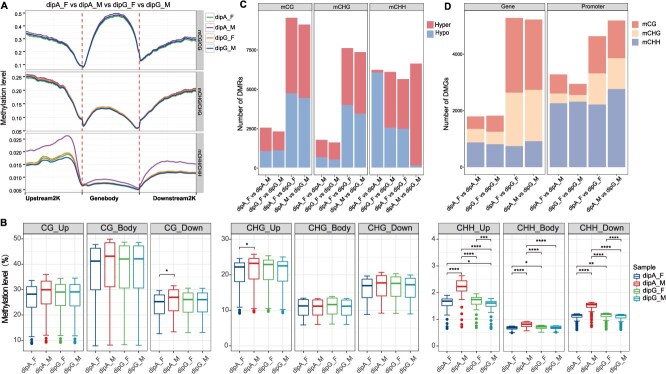
Differential methylation analysis among four floral buds of *C. paliurus*. (A) Methylation profiles in gene bodies and their respective 2-kb flanking regions. (B) Comparison of methylation levels in gene bodies and their upstream (Up) and downstream (Down) regions. Wilcoxon test, ^*^P < 0.05, ^**^P < 0.01, ^***^P < 0.001, ^****^P < 0.0001. (C) Counts of hypermethylated (Hyper) and hypomethylated (Hypo) context-specific DMRs in different comparisons. (D) Number of DMGs in gene body and promoter regions in different comparisons.

In this study, methylation profiles of gene-coding regions were analyzed extensively (Fig. 2A). From the flanking regions to the transcription start sites (TSS) and transcription end sites (TES), the methylation levels of CG and CHG contexts gradually decrease, while the methylation levels of CHH context first increase and then decrease (Fig. 2A). When comparing the methylation levels across various floral buds between two morphs, significant differences in methylation levels in protein-coding genes were observed in the downstream regions of CG sites and the upstream regions of CHG sites (Fig. 2B). Despite the lower CHH methylation levels, significant differences were detected among the four samples (Fig. 2B). Intramorph comparisons of CHH methylation levels within complementary sexual flowers revealed that dipA_M exhibited higher levels than dipA_F, and dipG_F surpassed dipG_M. Similarly, intermorph comparisons of same-sex flowers revealed higher CHH methylation levels in dipA_M compared to dipG_M, and higher levels in dipG_F compared to dipA_F (Figs 1C and 2B). These comparisons indicate that early-flowering buds (dipA_M and dipG_F) had higher CHH methylation levels than later ones (dipG_M and dipA_F), regardless of inter- or intramorph.

Furthermore, we conducted pairwise comparisons of DNA methylation profiles across four floral buds. For complementary sexual flowers of intramorph (i.e. dipA_F vs dipA_M and dipG_F vs dipG_M), 10 481 and 9955 differentially methylated regions (DMRs) were identified, respectively. While for congeneric flowers of intermorph (i.e. dipA_F vs dipG_F and dipA_M vs dipG_M), 22 691 and 23 000 DMRs were detected, respectively (Fig. 2C), in which a higher number of DMRs were observed in CG and CHG contexts. Intriguingly, many DMRs in the CHH context were exhibited in all comparisons. In dipA_F vs dipA_M, the number of hypomethylated (Hypo) DMRs (6035) was significantly higher than that of hypermethylated (Hyper) DMRs (164). An inverse pattern was observed in dipA_M vs dipG_M, where the number of Hypo-DMRs (128) was significantly lower than that of Hyper-DMRs (6468) (Fig. 2C). To better understand how DMRs affect genes, we defined DMR-associated genes (within the gene body) and DMR-associated promoters (2 kb upstream). Furthermore, the number of DMR-associated promoters at CHH sites exceeded 2000 in all comparisons (Fig. 2D). This is consistent with the significant differences observed between samples in the CHH context (Fig. 2B), suggesting that differential methylation at CHH sites predominantly occurs in promoter regions. Collectively, the most pronounced differences in CHH methylation levels were observed between male and female flowers of intramorph, with the early-flowering samples exhibiting higher methylation levels compared to the late-flowering ones.

### Association between DNA methylation and gene expression levels

**Figure 3:**
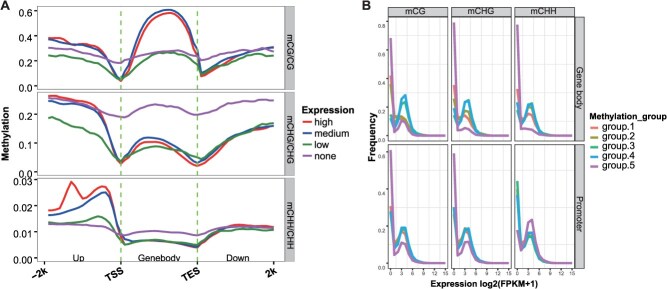
Relationship between DNA methylation and gene expression in *C. paliurus*. (A) Distribution of methylation levels in all three contexts within the gene body, upstream, and downstream regions based on four expression levels: none (FPKM <1), low (1 < FPKM <5), medium (5 < FPKM <25), and high (FPKM >25). (B) Expression profiles of methylated and unmethylated genes. Methylation genes were divided into five groups: Group 1 (bottom 20th percentile), Group 2 (20th–40th percentile), Group 3 (40th–60th percentile), Group 4 (60th–80th percentile), and Group 5 (top 20th percentile). Here, only the methylation level distribution and methylated and unmethylated gene expression profiles of dipA_F are shown, with the rest detailed in Fig. S4.

To explore the role of DNA methylation in gene expression regulation in *C. paliurus*, we performed transcriptome sequencing on identical materials. Three biological replicates showed high correlations (*R* > 0.85), with principal component 1 (PC1) and PC2 explaining 54.58% and 25.65% of the variation in gene expression, respectively (Fig. S3A). Over 1700 differentially expressed genes (DEGs) were identified between the two samples (Fig. S3B), suggesting distinct regulatory mechanisms in heterodichogamous *C. paliurus*. We then integrated methylome and transcriptome data. Genes were classified into four groups based on their fragments per kilobase of transcript per million mapped reads (FPKM) values: no expression, low, medium, and high. Plotting the methylation levels of the four expression groups, we observed consistent trends across different samples within the same sequence context (Fig. S4). In gene body regions, non-expressed genes exhibited elevated levels of CHG and CHH methylation compared to expressed genes (Figs 3A and S4). Conversely, highly expressed genes displayed increased methylation upstream of CG, CHG, and CHH sites, with the gene body having the highest levels of CG methylation (Figs 3A and S4). Additionally, upstream CHH methylation followed the trend: high > medium > low/none expression groups. Importantly, elevated upstream CHG methylation was observed in both non-expressed and highly expressed genes—highlighting the complexity of DNA methylation’s relationship with gene expression.

To investigate the correlation between DNA methylation and gene expression within various sequence contexts and regions of *C. paliurus*, we grouped genes into five sets. The grouping was determined by the methylation levels in the promoter and gene body regions. The results showed consistent gene expression trends across samples, suggesting that DNA methylation’s effect on gene expression is not tissue-specific (Fig. S4). In the gene body, Group 5 genes had the lowest expression levels at CG, CHG, and CHH sites, with no clear associations observed in other groups (Figs 3B and S4). In the promoter region, Group 5 genes had the lowest expression at CG and CHG sites but the highest expression at CHH sites, followed by Groups 4 and 3 (Figs 3B and S4). Overall, these findings point to a potential role of promoter CHH methylation in promoting gene expression in *C. paliurus*.

### Impact of methyltransferases and demethylases on DNA methylation in *C. paliurus*

**Figure 4:**
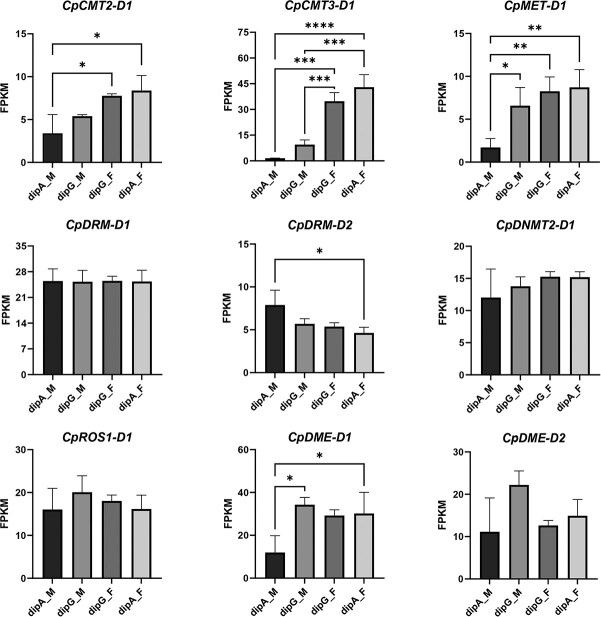
Transcript levels of DNA methyltransferase and demethylase genes among floral buds at Stage 3. Error bars represent the standard deviations of three biological replicates. Significance was determined by one-way ANOVA test, ^*^P < 0.05, ^**^P < 0.01, ^***^P < 0.001, ^****^P < 0.0001.

Six DNA methyltransferases and three demethylases have been identified from the diploid *C. paliurus* genome by our previous work [32]. The expression levels of DNA methyltransferases (*CpCMT2-D1*, *CpCMT3-D1*, *CpMET-D1*, and *CpDRM-D2*) and a demethylase (*CpDME-D1*) showed significant differences between male and female flowers in two morphs (Fig. 4, *P* < 0.05). Spearman correlation analysis revealed significant negative correlations between *CpMET-D1* and *CpDME-D1* with CHH methylation levels, as well as significant positive correlations between *CpDRM-D2* and both CHH and CG methylation levels (Fig. S5). The DNA methylation status can be dynamically regulated by the interplay of DNA methyltransferases and demethylases. Specifically, the reduced expression of *CpDME-D1* might lead to diminished demethylase activity, thereby elevating global DNA methylation levels, whereas the increased expression of *CpDRM-D2* could enhance methyltransferase activity, further promoting the establishment and maintenance of methylation at CHH sites. These coordinated alterations in the expression patterns of *CpDME-D1* and *CpDRM-D2* likely play a critical role in modulating sex-specific methylation states in male and female flowers of *C. paliurus*.

### Gene expression and methylation analysis in the flowering pathway of *C. paliurus*

**Figure 5:**
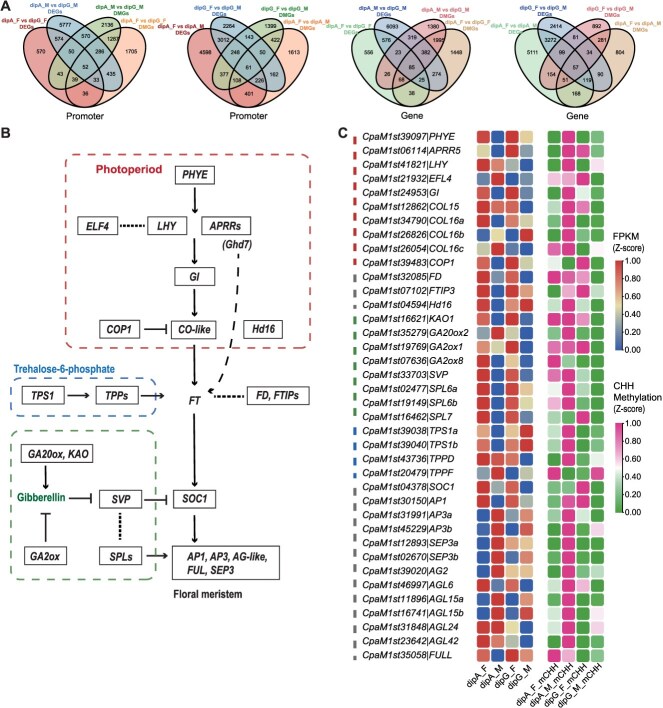
Expression and methylation analysis of genes related to the flowering pathway in *C. paliurus*. (A) Venn diagram of promoter-associated or gene-associated DMGs and DEGs in comparisons of floral organs. (B) MDEGs and putative schematic network of flowering induction pathways. (C) Heatmap of CHH methylation levels in the promoter regions and expression levels of flowering pathway genes. Gene expression or methylation levels for each gene were normalized by row.

The 748 upregulated and 649 downregulated genes were identified based on transcriptome sequencing comparison of dipA_F vs dipG_F, while about 6000–9000 DEGs were screened from other comparisons. Notably, the comparison of dipA_F vs dipA_M yielded a higher number of DEGs, consisting of 4210 upregulated and 4821 downregulated genes (Fig. S3B). To investigate epigenetic regulation underlying the heterodichogamous characteristics of *C. paliurus*, we identified overlapping promoters and genes between DMR-associated genes (DMGs) and DEGs in various comparisons. As shown in Fig. 5A, 160 overlapping promoters and 216 genes in dipA_F vs dipG_F and 958 overlapping promoters and 809 genes in dipA_M vs dipG_M, were identified, respectively. Correspondingly, 796 overlapping promoters and 395 genes in dipA_F vs dipA_M and 502 overlapping promoters and 271 genes in dipG_F vs dipG_M, were identified, respectively (Fig. 5A). These overlapping genes and promoters were collectively termed methylated-differentially expressed genes (MDEGs).

**Figure 6:**
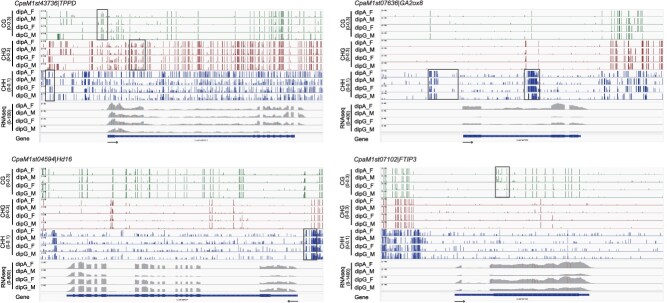
Integrative Genomics Viewer (IGV) displaying DNA methylation and gene expression profiles. The DNA methylation levels are indicated by the height of the vertical bars on each track. Black boxes indicate DMRs.

In this study, we identified several MDEGs associated with the flowering pathway, including the photoperiod pathway (e.g. *CpPHYE*, *CpLHY*, *CpELF4*, *CpGI*, and *CpHd16*), GA pathway (e.g. *CpGA20oxs*, *CpGA2oxs*, *CpSVP*, and *CpSPLs*), and trehalose-6-phosphate (Tre6P) signaling pathway (Fig. 5B). Among them, *CpLHY* showed significant differences in transcription levels between dipA_F vs dipG_F and dipA_M vs dipG_M. *CpHd16* expression was lower in the early-flowering samples compared to the late-flowering ones, specifically characterized by dipA_M < dipA_F and dipG_F < dipG_M (Fig. 5C). The expression levels of GA2ox family members were higher in dipA_F than in dipA_M, whereas the expression pattern of GA20ox was the opposite. In the Tre6P signaling pathway, *CpTPS1*, *CpTPPD*, and *CpTPPF* exhibited differential expressions between male flowers of the two morphs. The florigen-encoding gene *FT* serves as a central regulatory hub that coordinates multiple flowering pathways, interacting with *FTIP* and *FD* genes. The transcription levels of *CpFTIP3* and *CpFD* were higher in the female flowers than in the male flowers of both morphs, with dipG_M expression higher than dipA_M. Floral meristem identity genes *CpAP3*, *CpSEP3*, and *CpAG2* exhibited higher transcript levels in male flowers, particularly in the dipA_M, while *CpAP1* and *CpFULL* displayed higher transcript levels in female flowers across both morphs (Fig. 5C). These genes exhibited differential methylation levels in different sequence contexts or functional regions (Fig. 6). For example, *CpTPPD* had DMRs across three sequence contexts, including CHH and CG methylation differences in the promoter, and CHG methylation differences in the gene body. Similarly, *CpGA2ox8* exhibited CHH_DMRs in both the gene body and promoter regions. Based on the relationship between methylation levels and gene expression, we further examined the correlation between expression levels and promoter CHH methylation levels in flowering-related genes. *CpSVP* and *CpHd16* transcription levels were significantly negatively correlated with promoter methylation. In contrast, a significant positive correlation was observed for *CpAP3b*, *CpSEP3b*, *CpAG2*, and *CpCOL16c* (Figs 5C and S6). Therefore, we speculate that variations in methylation sites may be one of the causes of changes in gene expression levels, and further functional validation is still required.

### Overexpression of *CpHd16*, *CpFTIP3*, and *CpTPPD* delays flowering in *Arabidopsis*

**Figure 7:**
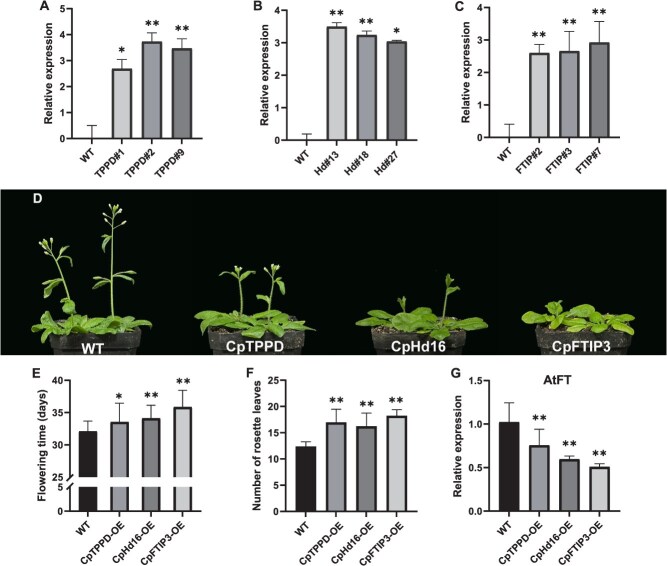
Heterologous overexpression of *CpTPPD*, *CpHd16*, and *CpFTIP3* delays flowering in *Arabidopsis*. (A–C) Relative expression levels of *CpTPPD* (A), *CpHd16* (B), and *CpFTIP3* (C) in three transgenic *Arabidopsis* lines, respectively. Relative expression levels were log10-transformed, and the mean ± SD was calculated from the log values. (*n* = 3, Wilcoxon test, ^*^P < 0.05, ^**^P < 0.01, comparing transgenic lines to WT) (D) Phenotype of transgenic plants overexpressing *CpTPPD*, *CpHd16*, *CpFTIP3*, as well as WT plants, was observed under long-day conditions for 30 days. (E) Flowering time of WT, *CpTPPD*, *CpHd16*, and *CpFTIP3* (data are mean ± SD, Wilcoxon test, ^*^P < 0.05, ^**^P < 0.01, comparing transgenic lines to WT, the same below). (F) Rosette number of WT and transgenic *Arabidopsis*. (G) Relative expression levels of *AtFT* gene in WT and transgenic *Arabidopsis*.

Based on changes in methylation levels and transcription levels, as well as the known functions of these genes in other species, we preliminarily selected three genes—*CpHd16*, *CpFTIP3*, and *CpTPPD*—for functional validation in the flowering pathway of *C. paliurus*. To investigate their roles, the coding sequences of these genes were heterologously overexpressed in Col-0 wild-type (WT) *Arabidopsis*. Transgenic plants were selected on Hygromycin B-containing medium, and transgene expression was validated by reverse transcription-quantitative polymerase chain reaction (RT-qPCR). A significant upregulation of 35S::*CpTPPD*, 35S::*CpHd16*, and 35S::*CpFTIP3* was successfully overexpressed in the corresponding lines, with significant differences compared to WT (Fig. 7A–C**)**. Phenotypic analysis of the three highest expressing lines (10–12 individuals per line) showed delayed flowering, with average delays of 1.5, 2, and 4 days for *CpTPPD*, *CpHd16*, and *CpFTIP3* overexpression, respectively, compared to WT (Fig. 7D and E). These results indicate that the overexpression of *CpHd16*, *CpFTIP3*, and *CpTPPD* affects flowering in *Arabidopsis* and may play similar roles in the regulation of flowering in *C. paliurus*. In agreement with the flowering phenotypes, the rosette leaf number at bolting was significantly increased in the transgenic plants. Specifically, the rosette leaf numbers in 35S::*CpTPPD*, 35S::*CpHd16*, and 35S::*CpFTIP3* transgenic *Arabidopsis* lines were increased by 4.5, 4, and 6 leaves, respectively, compared to WT (Fig. 7F). RT-qPCR analysis showed that, compared to WT, the expression levels of *AtFT* and *AtSOC1* were downregulated in the overexpression lines of above three genes (*CpTPPD*, *CpHd16*, and *CpFTIP3*), while that of *AtSVP* expression was upregulated (Figs 7G and S7). Additionally, the expression of *AtFD*, *AtSPL3*, and *AtAP1* was upregulated in *CpHd16*-OE lines, while *AtSPL4* was elevated in *CpTPPD*-OE lines (Fig. S7).

### 5-azaC inhibits floral development in *C. paliurus*

**Figure 8:**
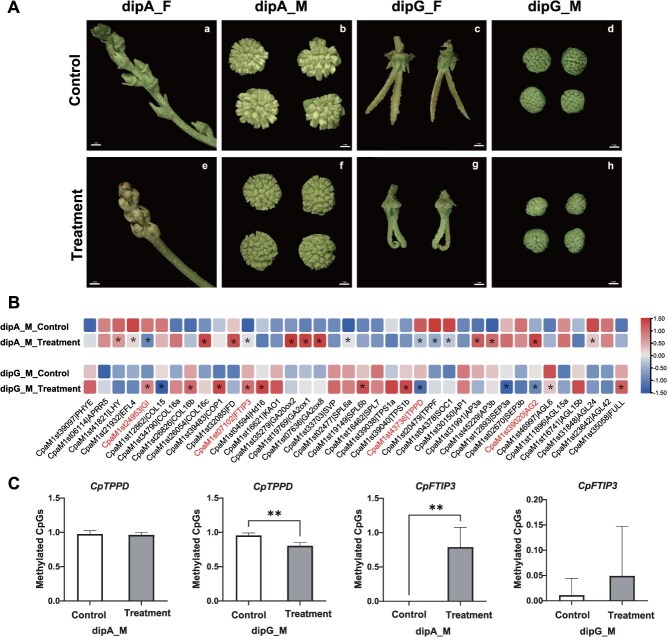
Effects of 5-azaC on flowering time and expression of related genes in *C. paliurus.* (A) Photographs of ddH_2_O (control) and 5-azaC treated *C. paliurus* at Stage 3. (a–d) Male and female floral buds of the two morphs in control groups. (e–h) Male and female floral buds of the two morphs in the treatment group. Scale bar, 1 mm. (B) Expression analysis of flowering pathway-related genes in control and 5-azaC treated male flowers of *C. paliurus*. (C) Bisulfite sequencing PCR analysis of methylation levels in *CpTPPD* promoter and *CpFTIP3* gene body. ^*^P < 0.05, ^**^P < 0.01: significant and extremely significant differences compared to the control group, respectively.

To validate the impact of DNA methylation on floral development, we applied 20 mM 5-azaC as a spray to the flowering branches of *C. paliurus* before the bud break stage. The treatment inhibited the development of both male and female flowers, accompanied by distinct phenotypic alterations (Fig. 8A). For instance, in protogyny, the growth of dipG_M under 5-azaC treatment was significantly hindered, with some male flowers on branches ceasing development (Fig. S8). Moreover, the treatment led to a higher count of female flowers, which exhibited varied developmental stages compared to the control group (Fig. S8). The 5-azaC treatment delayed the blooming of both dipA_F and dipA_M by 4–7 days (Fig. S9A). In protogyny, 5-azaC treatment delayed the blooming times for dipG_M by 2–6 days and for dipG_F by 2–5 days (Fig. S9B). Heatmap analysis of RNA-seq data revealed transcriptional changes induced by DNA hypomethylation in both male flower morphotypes (dipA_M and dipG_M), marked by significant suppression of *CpTPPD* and concurrent upregulation of *CpFTIP3* (Fig. 8B). Additionally, the transcription levels of some genes were only significantly different in dipA_M, including *CpLHY*, *CpFD*, *CpGA20ox2*, *CpGA2ox8*, and *CpGA2ox1* (Fig. 8B). The expression of *CpCOP1*, *CpHd16*, *CpSCL6b*, *CpTPS1b*, and *CpFULL* was upregulated exclusively in treated dipG_M (Fig. 8B). RT-qPCR analysis of eight randomly selected genes demonstrated expression trends concordant with transcriptome data (Fig. S10), validating the dataset’s reliability.

To further validate the effect of 5-azaC on the methylation levels of key flowering-related genes, we examined the methylation status of the target regions—derived from the DMRs identified via whole-genome bisulfite sequencing (WGBS)—in *CpTPPD* and *CpFTIP3* using bisulfite sequencing PCR (BSP). Consistent with the role of 5-azaC as a DNA methylation inhibitor, the methylation level of *CpTPPD* was decreased after treatment, whereas that of *CpFTIP3* was increased (Figs 8C and S11). These changes in methylation levels were consistent with alterations in their gene expression patterns. For instance, *CpTPPD* expression was downregulated in the treatment group (Fig. 8B), which further supported the regulatory role of DNA methylation in modulating the expression of key flowering-associated genes. Collectively, these results indicate that DNA methylation is crucial for the normal development of flowers in *C. paliurus*, and variations in genome-wide methylation levels may impact the transcription levels of genes involved in the flowering pathway.

## Discussion

Flowering represents a pivotal phase in the plant life cycle, which determines reproductive success and genetic diversity. Optimal flowering timing enhances seed yield and quality, which is particularly critical for perennial species with complex life-history strategies. Recent studies have demonstrated that DNA methylation modulates flowering response to vernalization and photoperiod by interacting with key flowering regulatory factors, such as *FLC*, *FT*, and *CO*, as well as plant hormones [8, 31, 33]. However, the role of DNA methylation in regulating heterodichogamy remains largely unexplored. In this study, we constructed single-base resolution methylomes for the male and female flowers across two morphs of diploid *C. paliurus*. Our findings provide the first evidence that dynamic DNA methylation patterns may play a regulatory role in the heterodichogamous flowering traits of this species.

Methylome analysis revealed that the methylation levels in flower organs of *C. paliurus* were highest in the CG context (47.59%–50.07%), followed by CHG (31.65%–32.54%) and CHH (1.35%–1.73%) (Fig. 1C), mirroring patterns observed in *Citrus*, *Malus domestica*, *D. glomerata*, *Salvia miltiorrhiza*, and *Phyllostachys edulis* [5, 23, 26, 34, 35]. For instance, in *Arabidopsis* (genome size 135 Mb), methylation levels in CG, CHG, and CHH contexts were ~ 30.5%, 9.3%, and 1.1%, respectively [36]. In *M. domestica* (742 Mb) floral buds, higher methylation levels for these contexts were reported, reaching 80%, 67.8%, and 23.7%, respectively [35]. As genome size increases, DNA methylation appears to rise correspondingly. Previous studies have shown a positive correlation between DNA methylation levels and genome size [26, 37, 38]. Diploid *C. paliurus*, with a genome size of ~580 Mb, exhibited relatively higher methylation levels compared to *Arabidopsis* and *Populus* (480 Mb) [36, 39]. However, when compared to *M. domestica* or *Beta vulgaris*, *C. paliurus* displayed notably lower methylation levels, especially in the CHH context [35]. These results indicate that different species or tissues exhibit distinct methylation patterns, and DNA methylation plays a crucial role in maintaining genomic stability.

In addition, DNA methylation influences gene expression, although the specific mechanisms in plants remain to be fully understood [3]. DNA methylation associated with genes is found in both promoter and gene bodies, and its complex relationship with gene expression makes it both a cause and an effect [40]. Typically, transcriptional repression is associated with promoter methylation, while gene body methylation shows a positive correlation with expression activation, as seen in rice and cotton [41, 42]. However, this pattern is not consistent across all species, as seen in *D. glomerata*, where the relationship is reversed [26]. In this study, we found that the majority of DEGs identified did not show a direct association with changes in DNA methylation (Fig. 5A). Among the MDEGs involved in the flowering pathway, only a subset was associated with methylation changes. CHH methylation alterations in the promoter affected gene expression, as shown in *CpAP3b*, *CpSEP3b*, *CpAG2*, and *CpHd16* (Fig. S6), while the relationship between gene body methylation and gene expression was more complex (Figs 3 and S4). These findings are consistent with those in *Arabidopsis* [40], indicating that the impact of DNA methylation on gene expression is relatively minor.

At the inflorescence elongation stage of *C. paliurus*, early-flowering samples exhibited higher methylation levels than the late-flowering ones, with particularly significant differences observed in CHH contexts (Figs 1C and 2B). The increase in DNA methylation levels could result from either enhanced activity of DNA methyltransferases or diminished activity of demethylases [43]. In this study, we found that in the earliest flowering sample dipA_M, the methyltransferase gene *CpDRM-D2* was highly expressed (Fig. 4), potentially catalyzing CHH methylation and maintaining genomic hypermethylation. In contrast, the expression of demethylase *CpDME-D1* was downregulated (Fig. 4), and its reduced activity further contributed to the hypermethylated state. These findings are consistent with observations reported in the floral organs of lotus (*Nelumbo nucifera*) [44]. Moreover, the transcriptional abundance of *CpDRM-D2* and *CpDME-D1* was significantly correlated with CHH methylation (Fig. S5), suggesting that the CHH hypermethylation observed during floral development in *C. paliurus* may be dynamically regulated by the interplay between *CpDRM-D2* and *CpDME-D1*. The dynamic changes in CHH methylation are an important pattern in plant flowering, which can promote flowering during vernalization in *D. glomerata* through hypermethylation and in *P. edulis* through hypomethylation [23, 26]. These findings highlight the critical role of DNA methylation in regulating flowering time and suggest that the interplay between methyltransferases and demethylases is a key factor in this process.

Characterizing MDEGs between male and female flowers of two morphs in *C. paliurus* provides critical insights into the molecular regulatory mechanisms underlying heterodichogamy. Our analysis identified several MDEGs implicated in key flowering pathways, including photoperiod, GA, and Tre6P signaling pathways (Fig. 5B). Among the candidate genes, *CpHd16*, which shares sequence homology with the rice *OsHd16/EL1* gene, exhibited a negative correlation between its transcript levels and CHH methylation levels in the promoter region (Fig. S6). In intramorph comparisons, *CpHd16* expression was downregulated in the early-flowering samples compared to the late-flowering ones (Fig. 5C). In rice, the *OsHd16/EL1* gene delayed flowering by phosphorylating *Ghd7* to enhance the photoperiodic response [45]. Functional validation in transgenic *Arabidopsis* revealed that *CpHd16* overexpression delayed flowering, accompanied by a marked reduction in *AtFT* transcript levels and a notable increase in *AtSVP* expression (Figs 7and S7). *SVP* has been demonstrated to directly bind the *FT* promoter and repress its expression [46], indicating disruption of florigen synthesis signaling and strongly supporting the role of *CpHd16* as a potential flowering repressor in *C. paliurus*. Similarly, the delayed flowering phenotype observed in *CpFTIP3*-OE lines was also accompanied by a decrease in *AtFT* expression (Fig. 7). *CpFTIP3* exhibited differential expression between two morphs and distinct CG methylation patterns across gene body (Figs 5 and 6). After 5-azaC treatment, both the transcriptional and methylation levels of *CpFTIP3* were higher than those in the control group (Fig. 8C). This expression pattern is consistent with the observed higher abundance of *CpFTIP3* in late-flowering samples compared to early-flowering ones (Fig. 5C). FTIPs mediate the nuclear import of FT protein and play crucial roles in plant development, yet the functions of most FTIPs remain unknown [47]. For instance, *FTIP1* regulated flowering time in both *Arabidopsis* and rice [48, 49]; *FTIP3/4* were essential for shoot meristem development in *Arabidopsis* [50]; and *OsFTIP7* controlled anther dehiscence in rice [51]. Previous research identified three *FTIP1* genes associated with flowering-related QTLs in *J. regia* [52]. These findings highlight that FTIPs may play a central role in regulating flowering time in heterodichogamous plants.

The Tre6P signaling pathway integrates environmental and internal physiological signals to regulate flowering. In this study, genes involved in the Tre6P signaling pathway, such as *CpTPS*, *CpTPPD*, and *CpTPPF*, exhibited differences in both expression and methylation levels between different flower buds (Figs 5C and 6). During the inflorescence elongation stage, *CpTPPD* expression was higher in early-flowering dipA_M than in late-flowering dipG_M (Fig. 5C), while the reverse pattern was observed during bud break, with higher *CpTPPD* expression in dipG_M (Fig. S12), suggesting that *CpTPPD* expression at bud break may contribute to delayed flowering. This aligns with recent observations in *Juglans*, where *TPPD1* is highly expressed in protogynous male flowers during early floral development, and its single locus governs heterodichogamy [18], further supporting its conserved role in delaying floral development. Heterologous overexpression of *CpTPPD* in *Arabidopsis* delayed flowering and perturbed the expression of *AtFT* (Fig. 7), a central integrator of flowering pathways. Since Tre6P promoted flowering by regulating *FT* expression in leaf phloem companion cells [53]. We speculate that *CpTPPD* overexpression may reduce Tre6P levels, thereby disrupting the normal regulation of flowering by the Tre6P signaling pathway and ultimately leading to delayed flowering. In addition, 5-azaC treatment delayed flowering time in *C. paliurus* and reduced both the methylation level in the *CpTPPD* promoter region and its transcriptional level during the inflorescence elongation stage (Figs 8 and S9). We hypothesize that heterodichogamy in *C. paliurus* may be influenced by the combined effects of DNA methylation and a polygenic regulatory network, with *CpTPPD* and *CpFTIP3* potentially mediating floral transition through methylation-dependent regulation. The flowering regulatory network of *C. paliurus* is currently mapped primarily based on known pathways in model plants, but its epigenetic molecular basis remains underexplored. In future studies, we plan to combine chromatin immunoprecipitation PCR (ChIP-PCR) with methylation editing techniques to directly manipulate the methylation levels of key loci, verifying whether methylation changes causally underlie the observed gene expression shifts.

## Materials and methods

### Plant materials and sample collection

Diploid *C. paliurus* (2*n* = 2*x* = 32) grows in Dashishan, Liyang, Jiangsu Province (119°48′E, 31°42′N), which is at the stage of vigorous reproduction. Nine well-growing individuals for two morphs, protogyny and protandry, were selected for observation and sampling. Following the research of Chen *et al*. [54], floral bud differentiation and development, benchmarked against the male flowers of protandry, five stages were identified: physiological differentiation (Stage 0), dormant period (Stage 1), bud break (Stage 2), inflorescence elongation (Stage 3), and mature (Stage 4). Since the difficulty in morphological distinction for female buds of *C. paliurus* during Stages 0–2, both male and female floral buds at Stage 3 were collected on 14 April 2023. The female and male flowers from protandry are designated as dipA_F and dipA_M, respectively, while those from protogyny are designated as dipG_F and dipG_M. Each sample with three biological replicates, including three individuals, was collected and immediately stored in an ultralow temperature freezer until DNA and RNA extraction.

### Whole-genome bisulfite sequencing

Genomic DNA was extracted from dipA_F, dipA_M, dipG_F, and dipG_M using the Magnetic Plant Genomic DNA Kit (Tiangen, Beijing, China). DNA quantification and integrity were evaluated using a Qubit Fluorometer (Qubit, Invitrogen, USA) and electrophoresis on 1% agarose gel. To establish analytical controls, Lambda phage DNA was used as a negative reference. DNA samples were mechanically sheared to generate 200- to 300-bp fragments using a Covaris S220 focused-ultrasonicator (Covaris, USA). Subsequently, bisulfite conversion was performed on single-stranded DNA fragments, which were then subjected to bisulfite treatment and used to construct DNA molecules for methylome analysis. Library preparation was carried out with the Accel-NGS® Methyl-Seq DNA Library Kit (Swift Biosciences, USA, Cat. No. 30096), followed by quality control assessment on the Agilent 5400 Bioanalyzer system (Agilent, USA). Normalized libraries at 1.5 nM concentration were quantified via qPCR before undergoing 150-bp paired-end sequencing on an Illumina NovaSeq 6000 platform (Illumina, USA). Technical support was provided by Beijing Novogene Bioinformatics Technology Co., Ltd. (Beijing, China).

### Whole-genome bisulfite sequencing data analysis

Raw sequencing data were initially assessed for quality using FastQC, followed by the removal of adapter sequences and low-quality bases (Phred score ≤ 20). Bisulfite-treated reads were aligned to the diploid genome of *C. paliurus* (GWH: GWHBKKX00000000, [10]) using Bismark [55]. Prior to alignment, the reference genome of diploid *C. paliurus* was computationally converted to simulate bisulfite treatment (C to T and G to A) and indexed using bowtie2 [56]. Processed sequencing reads underwent *in silico* conversion before being directionally mapped to this modified reference. Methylation status was subsequently determined by comparing both conversion orientations against the original genome sequence. Cytosine methylation status was determined using a statistical model incorporating methylated cytosine counts (mC), total cytosine counts (mC + umC), and nonconversion rate. Significant methylation sites were identified through binomial testing with a false discovery rate (FDR)-corrected *P*-value <0.05. For regional analysis, the genome was partitioned into 10-kb contiguous windows, where methylation levels were calculated as the percentage of mC among total cytosines at each genomic interval. Following alignment and methylation state inference, the average methylation levels across gene bodies, TEs, and the 2000-bp flanking regions upstream and downstream were calculated for three sequence contexts (CG, CHG, and CHH).

Detection of DMRs was performed using the DSS analysis software, which employed a sliding window approach with a window size of 200 bp and a step size of 50 bp, applying a conservative significance cut-off (*P* ≤ 1 × 10^−5^) [57, 58]. When the number of DMRs in comparisons exceeded 10 000, the first 10 000 were selected for subsequent analysis. Genes exhibiting DMRs within their transcriptional units or putative regulatory sequences (2 kb upstream of TSS) were classified as DMGs.

### RNA extraction and RNA-seq analysis

Total RNA was extracted from each sample using the E.Z.N.A.® Plant RNA Kit (Omega, Atlanta, Georgia, USA). RNA quality was assessed using the NanoDrop 2000 Spectrophotometer (Thermo Scientific, Waltham, MA, USA). RNA integrity was evaluated with the RNA Nano 6000 Assay Kit on the Agilent Bioanalyzer 5400 system (Agilent Technologies, CA, USA). RNA-seq libraries were constructed and sequenced on the Illumina NovaSeq 6000 platform (Illumina, USA) by Beijing Novogene Bioinformatics Technology Co., Ltd. (Beijing, China). The raw sequencing data have been deposited in the Genome Sequence Archive (GSA) database under Project Accession No. PRJCA030315.

To ensure quality and reliability, we filtered out reads containing adapter sequences and those with low Phred quality scores (*q* ≤ 5). The clean reads were aligned to the diploid *C. paliurus* genome assembly using HISAT2 [59]. Transcript abundance was quantified using the featureCounts, followed by FPKM normalization to enable cross-sample comparison of expression profiles [60]. Differential expression analysis was performed using the DESeq2 package [61]. DEGs were identified based on comparisons that met the criteria of |log2(FoldChange)| ≥ 1 and an adjusted *P*-value (FDR) ≤ 0.05.

### Overexpression of *CpHd16*, *CpFTIP3*, and *CpTPPD*

The full-length sequences of *CpHd16* (CpaM1st04594) and *CpFTIP3* (CpaM1st07102) were inserted into a pCambia1305 vector, while *CpTPPD* (CpaM1st43736) was inserted into a pCambia1302. All constructs contained the CaMV 35S promoter for constitutive expression. *Arabidopsis* (Col-0 ecotype) was transformed via the floral dip transformation performed using *Agrobacterium tumefaciens* GV3101. T1 generation was selected on half-strength Murashige and Skoog (MS) medium containing 30 μg/ml hygromycin B. Putative transgenic lines were confirmed through PCR amplification of transgenes using gene-specific primers (Table **S2**). Transgenic *Arabidopsis* and WT *Arabidopsis* were grown under a 16-h light/8-h dark photoperiod at a constant temperature, and flowering time was recorded.

### Reverse transcription-quantitative PCR

Primers for target genes and internal reference genes (*Atactin-2* for *Arabidopsis* and *Cp18S* for *C. paliurus*) were designed using Primer Premier 5.0 (PREMIER Biosoft, Canada), with amplicon lengths ranging from 100 to 300 bp (Table **S2**). RT-qPCR was conducted using the SYBR Green Master Mix (StepOnePlus System, Thermo Fisher Scientific). Relative expression levels were determined using the 2^-∆∆CT^ method with three biological replicates [54].

### Methylation inhibitor treatment and RNA-seq

From mid-March to early April of 2024, the above-selected individuals were treated with the methylation inhibitor 5-azacitidine (5-azaC, CAS 320–67-2). The 20 mM 5-azaC solubilized in ddH_2_O with 0.01% (v/v) Triton X-100 (CAS 9002-93-1) was sprayed onto the flowering branch (as treatment). As a control, other individuals were sprayed with ddH_2_O containing 0.01% Triton X-100 without 5-azaC. The spraying treatment was conducted every 7–10 days, totaling four times. At Stage 3 (21 April 2024), male and female flowers of two morphs were collected from control and treatment groups. Samples were stored in an ultralow-temperature freezer until further use. Meanwhile, the morphological characteristics of the floral buds were documented. RNA was extracted from dipA_M and dipG_M samples from both the control and treatment groups for transcriptome sequencing.

### Bisulfite sequencing PCR analysis

Genomic DNA was extracted from samples of the 5-azaC treatment group and control group using the TSINGKE Plant Genomic DNA Extraction Kit (TSP101). The extracted DNA was subjected to bisulfite conversion using the EpiTect Fast DNA Bisulfite Kit (Cat. No. 59824, Qiagen). Primers were designed to amplify the CpG island fragments of target regions using Methyl Primer Express v1.0 software, followed by nested PCR amplification with TSINGKE 2× Master Mix (blue). Detailed primer information is provided in Table S2. PCR amplicons were purified via gel extraction, then ligated into the T-vector using the pClone007 Versatile Simple Vector Kit (TSV-007VS). The ligation products were transformed into competent cells, which were then plated on selective agar medium. Ten single bacterial colonies were picked from each plate and sent to TSINGKE Biological Technology Co., Ltd. for Sanger sequencing.

### Statistical analysis

Pairwise group comparisons were conducted through nonparametric Wilcoxon rank-sum tests in GraphPad Prism 10. For multiple group comparisons, one-way ANOVA was performed using R version 4.3.1. Additionally, bivariate associations were assessed via Spearman correlation in R version 4.3.1.

## Supplementary Material

Web_Material_uhaf296

## Data Availability

The reference genome of diploid *C. paliurus* is available in the Genome Warehouse (GWH, CNCB; https://ngdc.cncb.ac.cn/gwh/Assembly/26382/show) [10]. Whole-genome bisulfite and transcriptome sequencing data of female and male flower buds (both morphs) at the inflorescence elongation stage are deposited in the Genome Sequence Archive (GSA) under accessions PRJCA040703 and PRJCA030315, respectively. Transcriptome data of 5-azaC-treated male flowers (both morphs) of diploid *C. paliurus* are deposited in GSA under PRJCA045638.
