# Supplementary material for: Differential methylation analysis of floral buds between two morphs unravels the contributions of key genes to flowering time in heterodichogamous Cyclocarya paliurus
Source: Hortic Res. 2025 Nov 3;13(2):uhaf296. doi: 10.1093/hr/uhaf296 (PMC12923269; doi:10.1093/hr/uhaf296)
Supplement: Web_Material_uhaf296 [file web_material_uhaf296.zip › Supplemental Figures.docx]

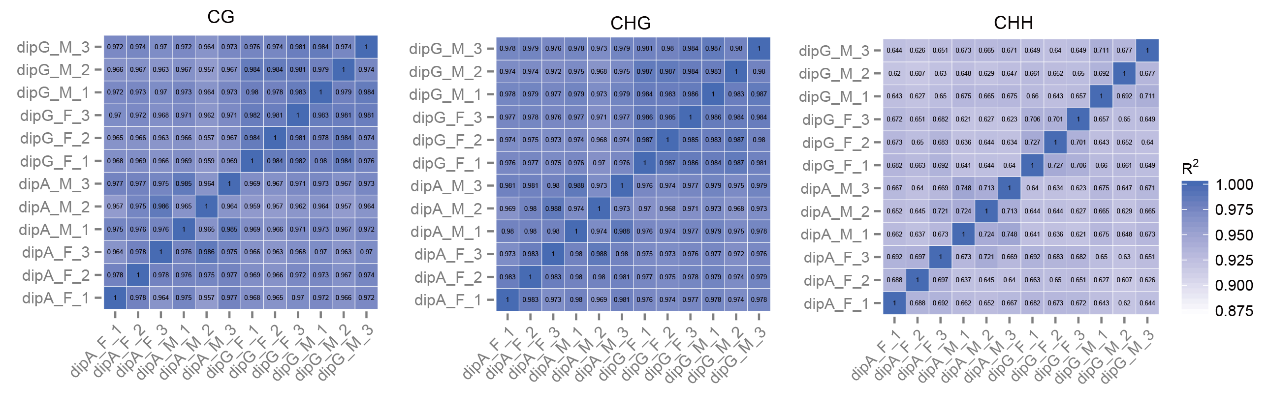


**Supplemental Figure S1.** Correlation of methylation levels in CG, CHG, and CHH contexts among samples


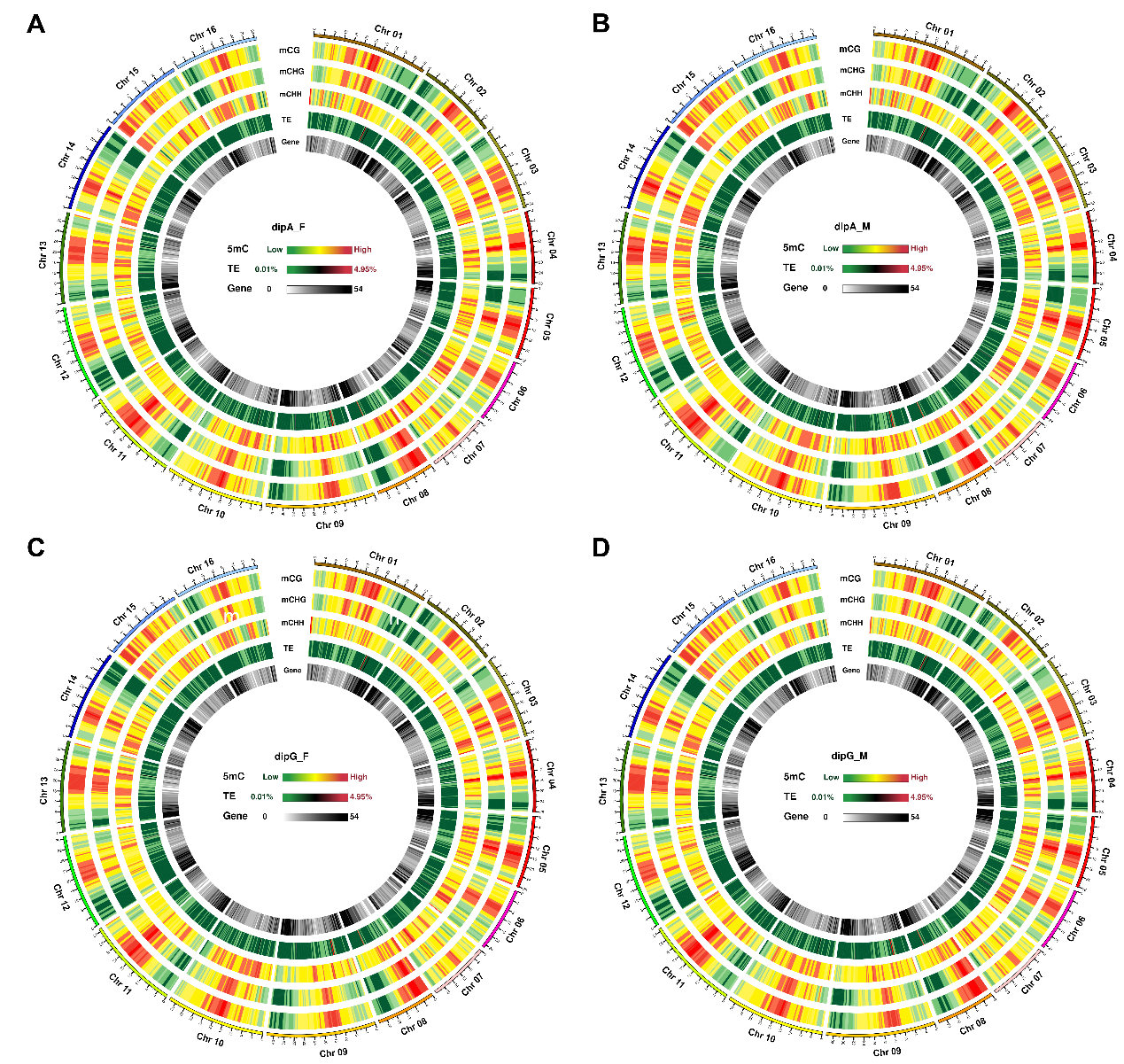


**Supplemental Figure S2.** Chromosome density circle diagrams of CG/CHG/CHH sequence contexts, transposable elements (TEs), and gene density in dipA_F (A), dipA_M (B), dipG_F (C), and dipG_M (D), respectively.


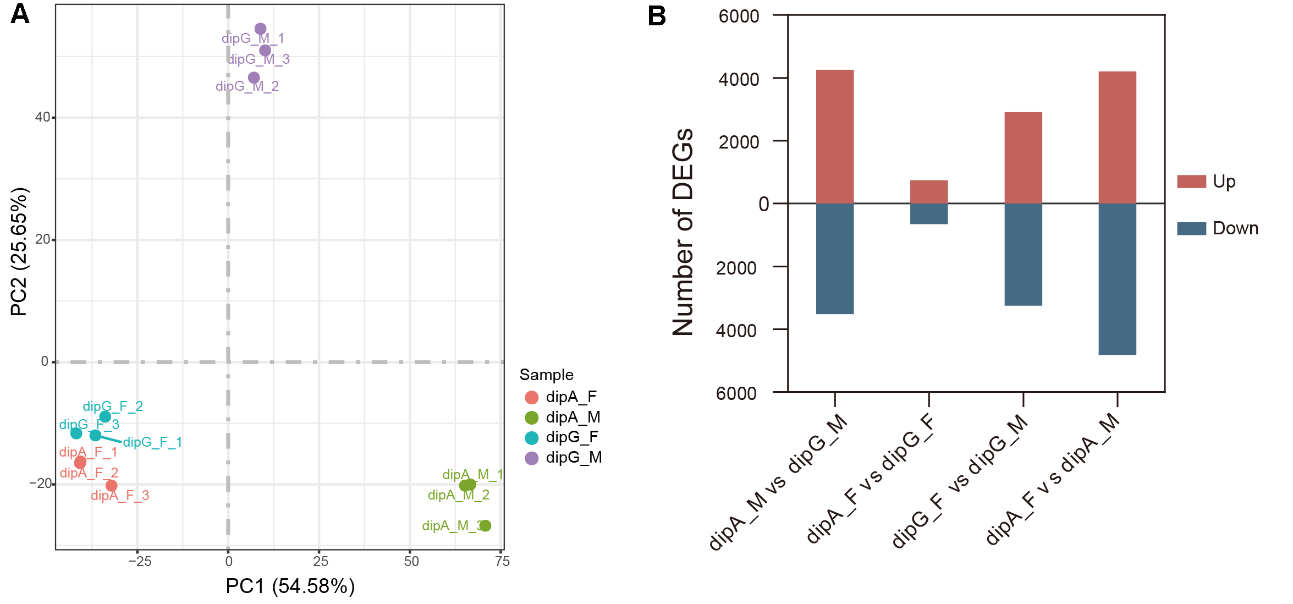


**Supplemental Figure S3.** Transcriptomic profiling of floral buds in *C. paliurus*. (A) Principal component analysis (PCA) of RNA-seq data across 12 floral samples. (B) Number of DEGs identified from different comparisons.


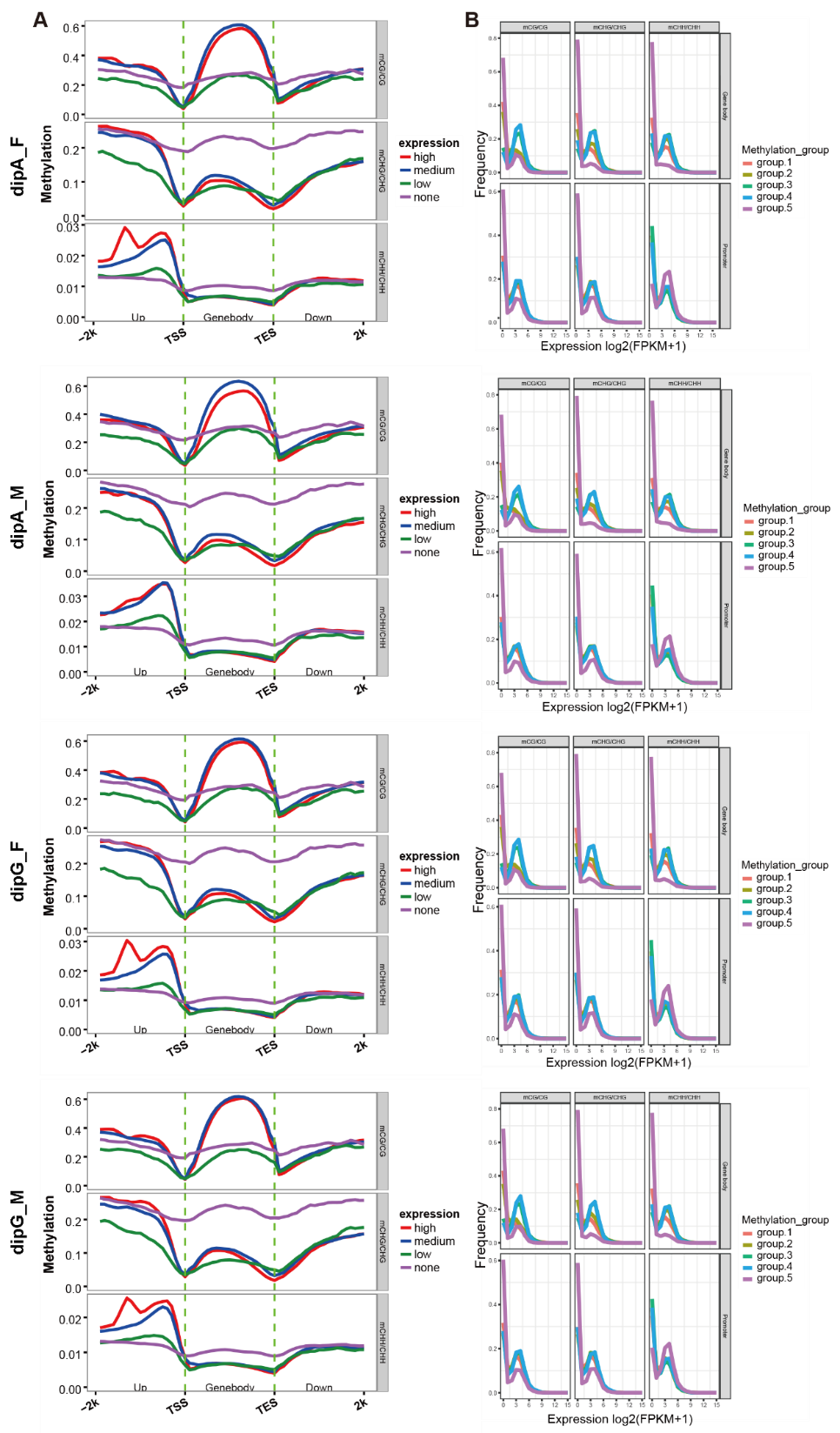


**Supplemental Figure S4.** Relationship between DNA methylation and gene expression in *C. paliurus*. (A) Distributions of methylation levels in three contexts within the gene body, upstream, and downstream regions based on four expression levels: none (FPKM < 1), low (1 < FPKM < low quartile), medium (low quartile < FPKM < upper quartile), and high (FPKM > upper quartile). TSS, the transcription start sites; TES, the transcription end sites. (B), Expression profiles of methylated and unmethylated genes. Methylation genes were divided into five groups: group 1 (< low quintile), group 2 (low quintile to second quintile), group 3 (the third quintile to fourth quintile), group 4 (the fourth quintile to upper quintile), and group 5 (> upper quintile).





**Supplemental Figure S5.** Correlation analysis between DNA methylation and the expression levels of DNA methyltransferase and demethylase genes.


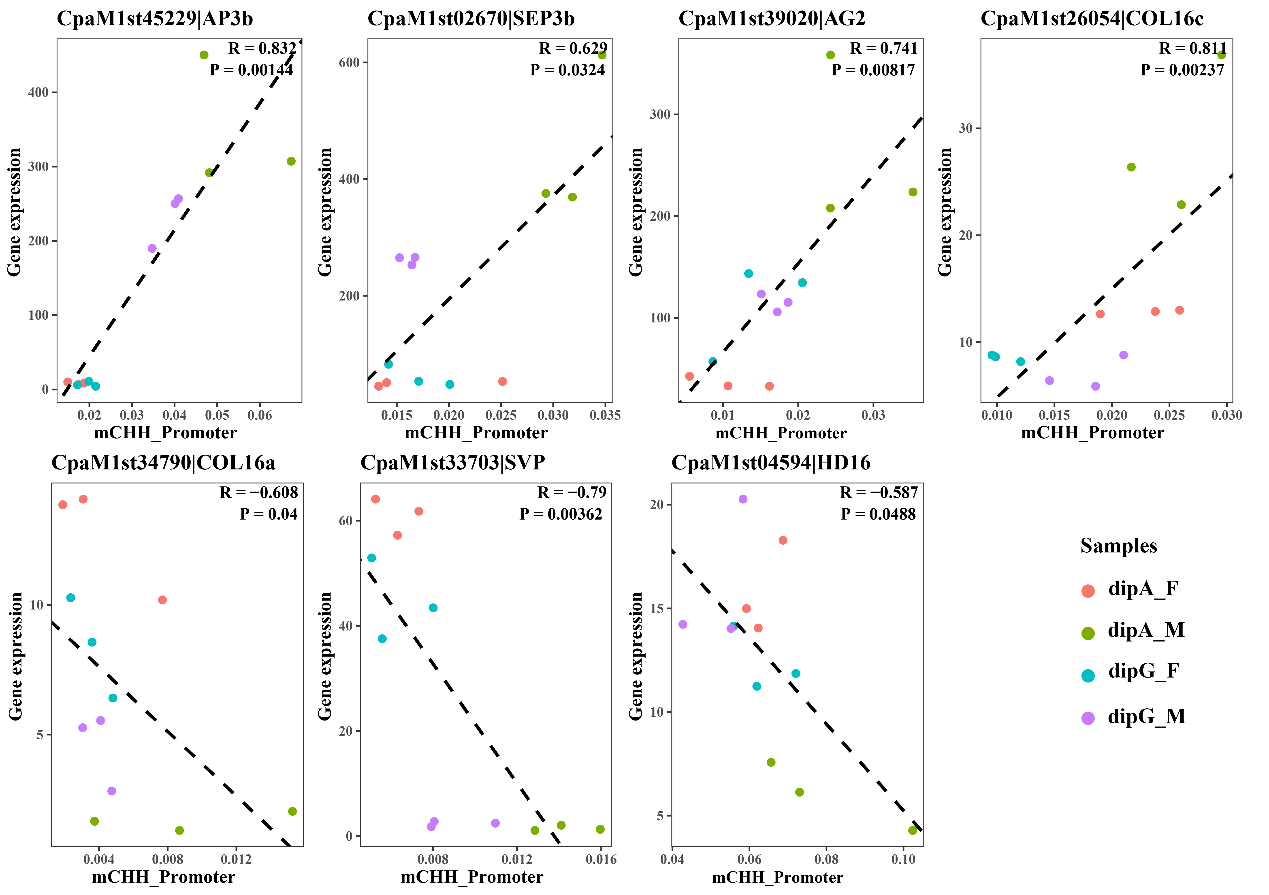


**Supplemental Figure S6.** Correlation analysis between the CHH methylation levels and expression levels in flowering pathway genes.


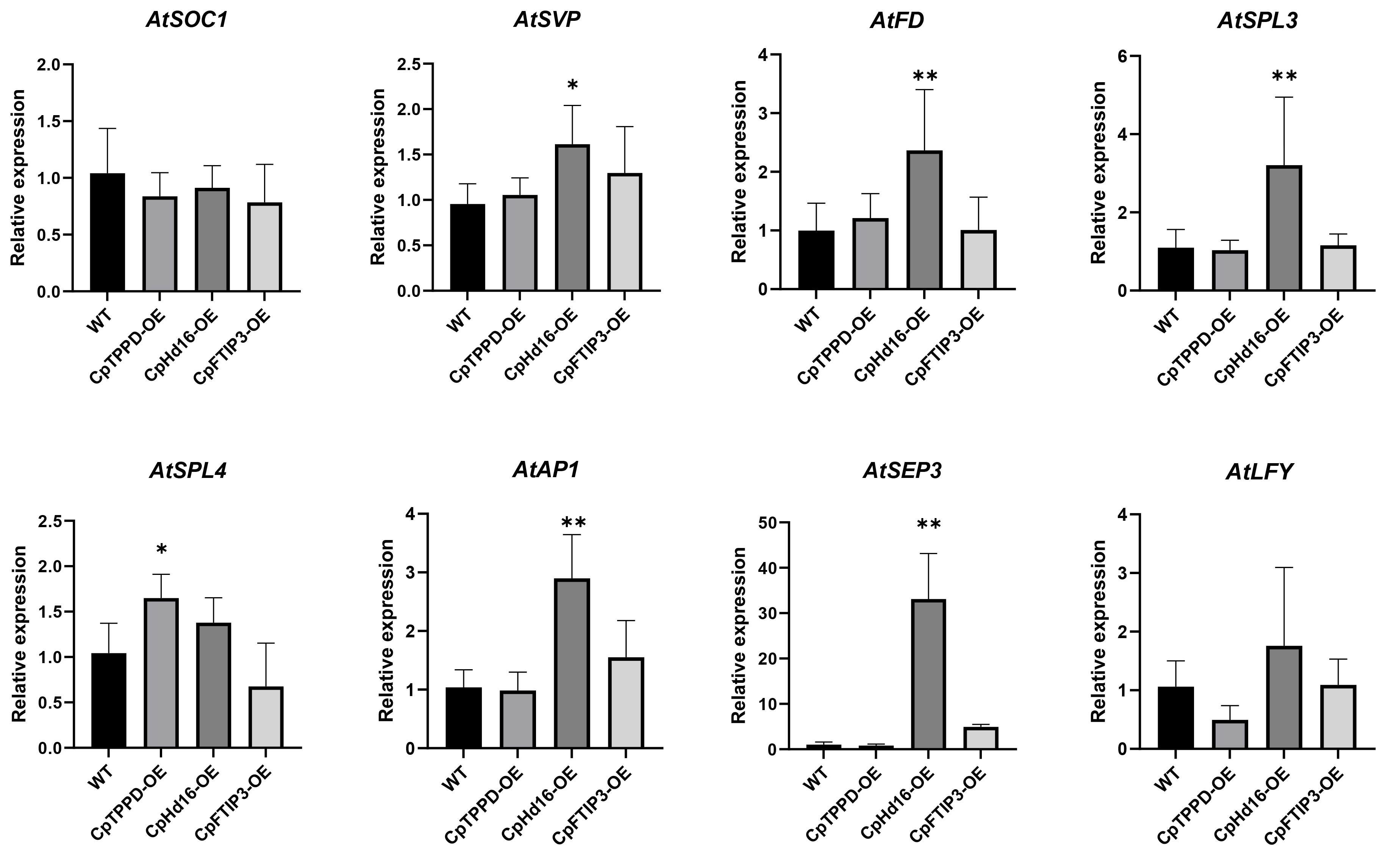


**Supplemental Figure S7.** Relative expression levels of flowering-related genes in wild-type (WT) and transgenic *Arabidopsis*. Data are mean ± SD, Wilcoxon test: **P* < 0.05, ***P* < 0.01 vs. WT.


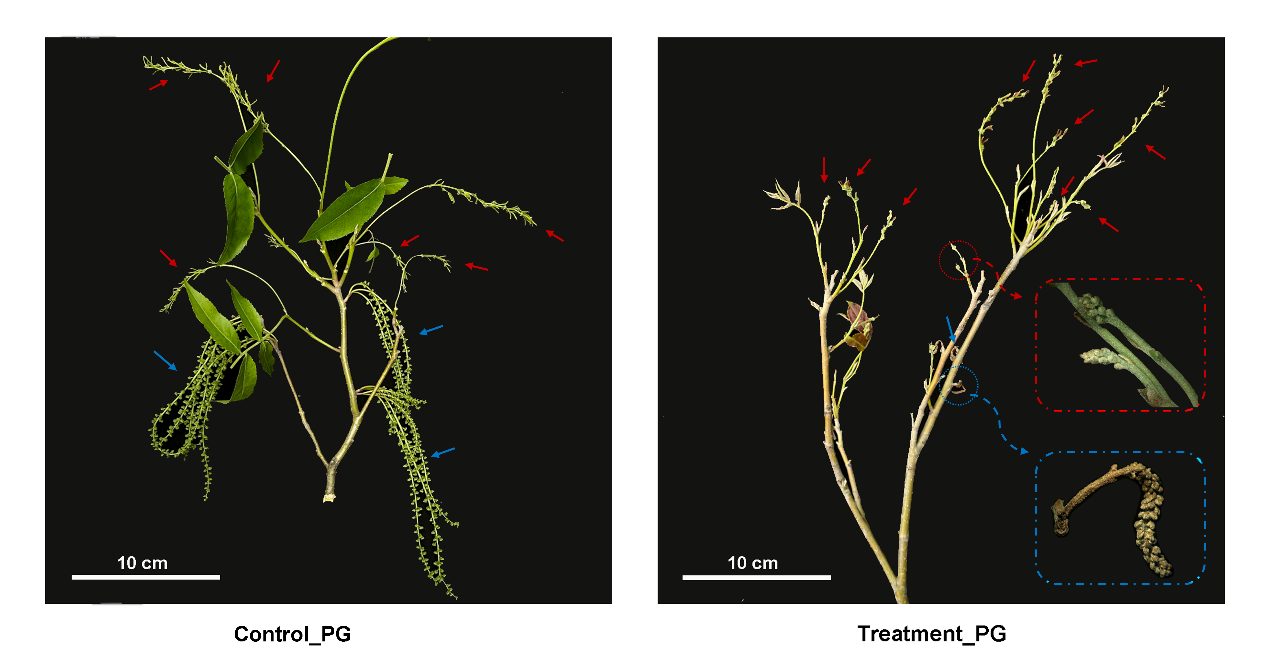


**Supplemental Figure S8.** The effect of 5-azaC treatment on the flowering of protogyny *C. paliurus*. Scale bar, 10cm; red arrows, indicate the female flowers; blue arrows, represent the male flowers.


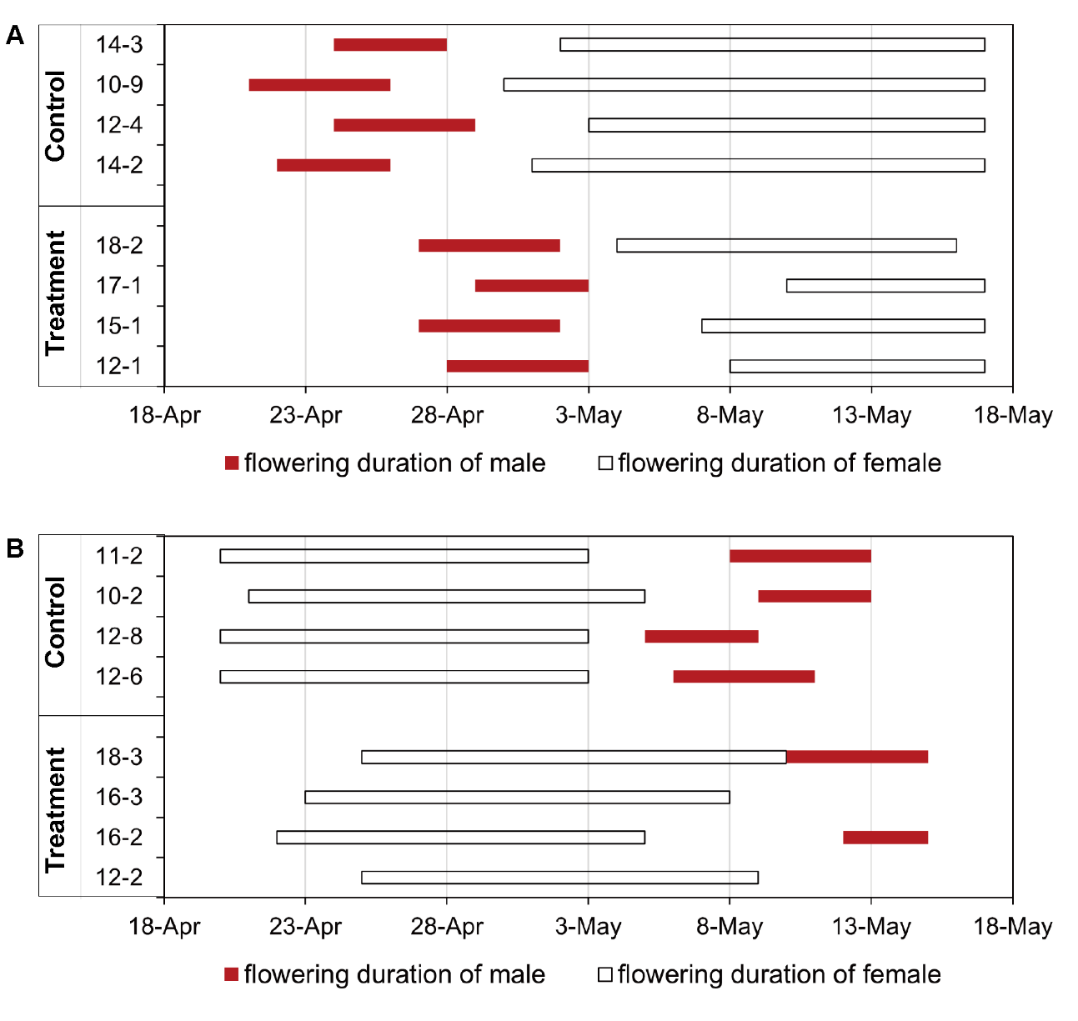


**Supplemental Figure S9.** Phenological changes in the flowering of male and female flowers of *C. paliurus* after 5-azaC treatment in protandry (A) and protogyny (B).


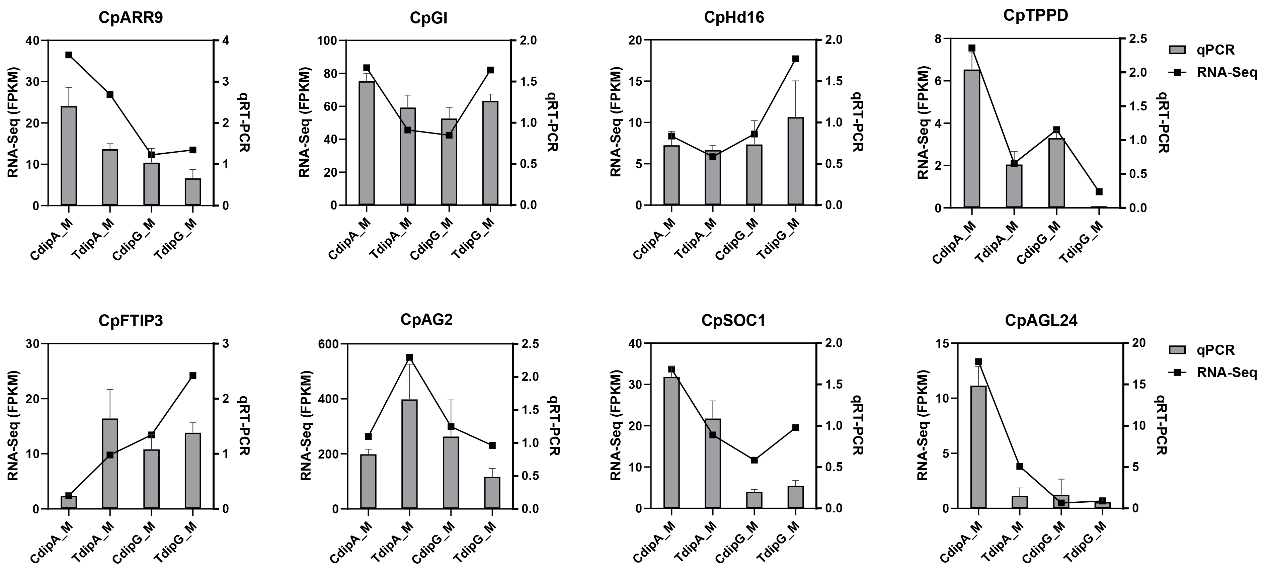


**Supplemental Figure S10.** Expression levels of flowering-related genes detected by RNA-seq and RT-qPCR.


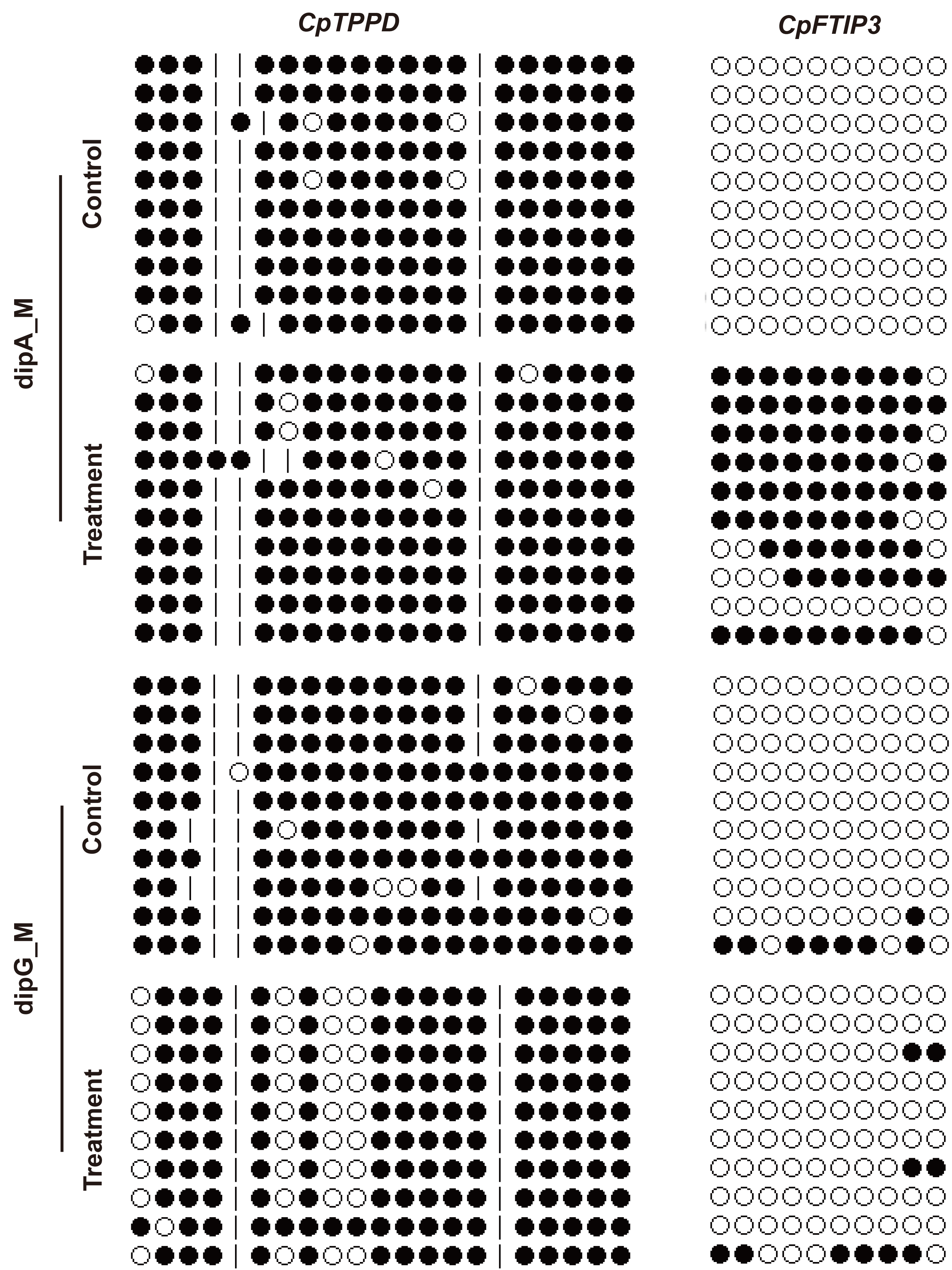


**Supplemental Figure S11.** DNA methylation patterns of CpG island in the *CpTPPD* promoter and *CpFTIP* gene body regions. Bisulfite sequencing PCR results from dipA_M and dipG_M samples. Each row represents an individual cloned sequence; each column corresponds to a single CpG site within the CpG islands. Open and filled circles indicate unmethylated and methylated cytosines, respectively.


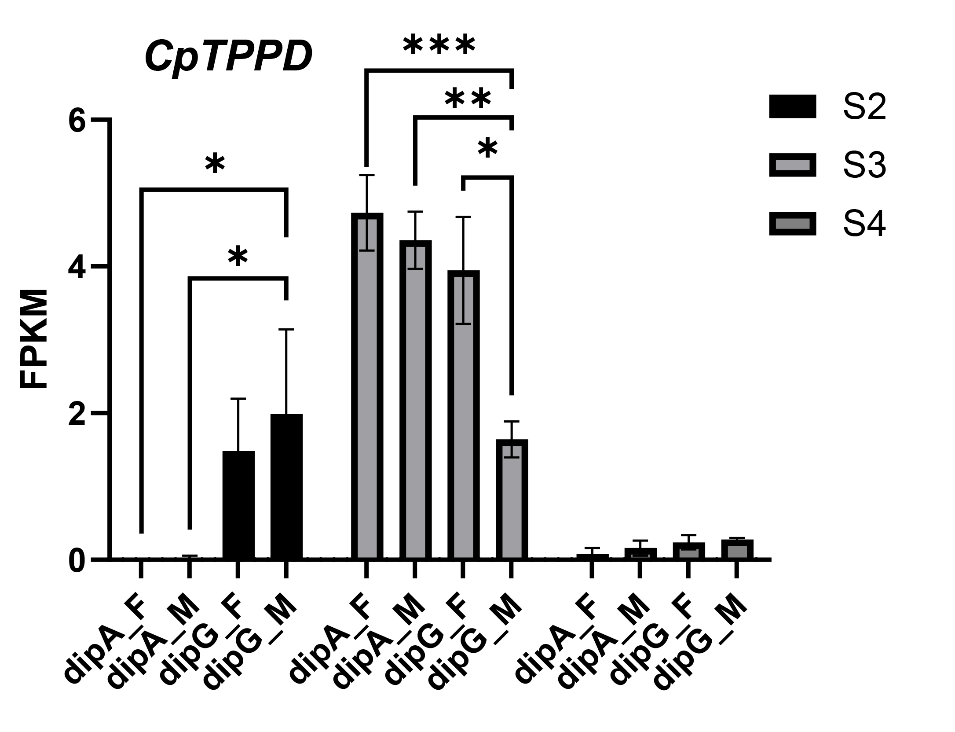


**Supplemental Figure S12.** Expression levels of *CpTPPD* in female and male flowers of *C. paliurus* across developmental stages. S1, bud break stage; S2, inflorescence elongation stage; S3, mature stage.
