# Supplementary material for: Differential methylation analysis of floral buds between two morphs unravels the contributions of key genes to flowering time in heterodichogamous Cyclocarya paliurus
Source: Hortic Res. 2025 Nov 3;13(2):uhaf296. doi: 10.1093/hr/uhaf296 (PMC12923269; doi:10.1093/hr/uhaf296)
Supplement: Web_Material_uhaf296 [file web_material_uhaf296.zip › Supplemental Materials.docx]

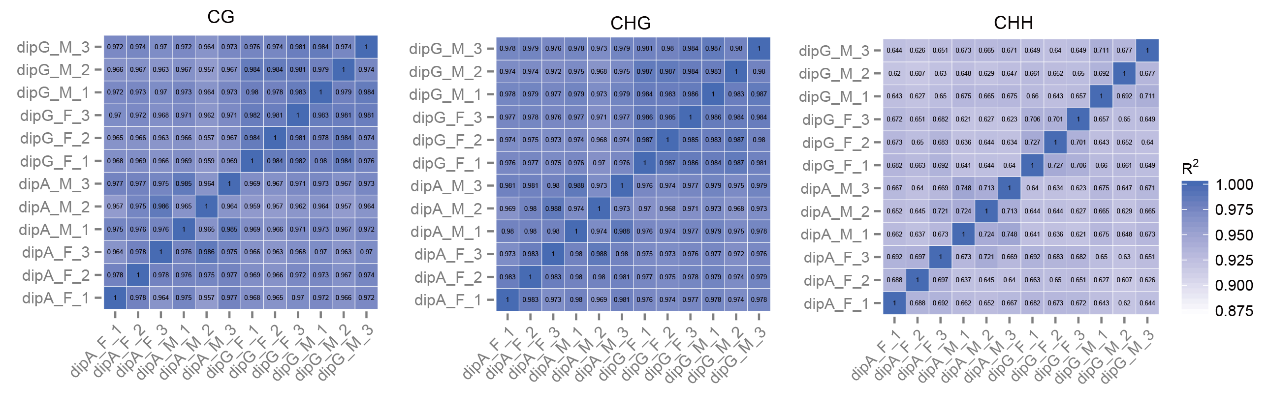


**Supplemental Figure S1.** Correlation of methylation levels in CG, CHG, and CHH contexts among samples


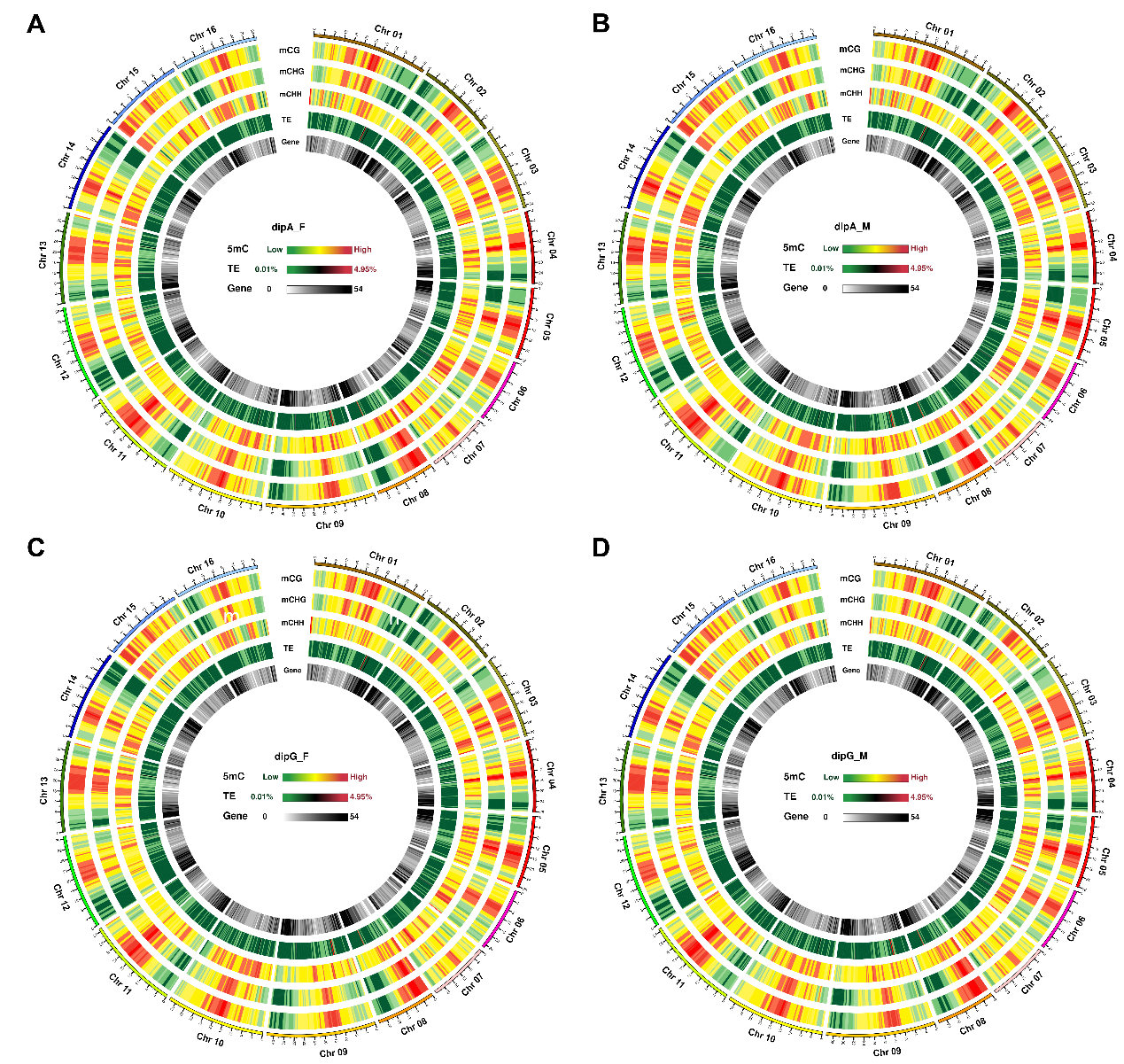


**Supplemental Figure S2.** Chromosome density circle diagrams of CG/CHG/CHH sequence contexts, transposable elements (TEs), and gene density in dipA_F (A), dipA_M (B), dipG_F (C), and dipG_M (D), respectively.


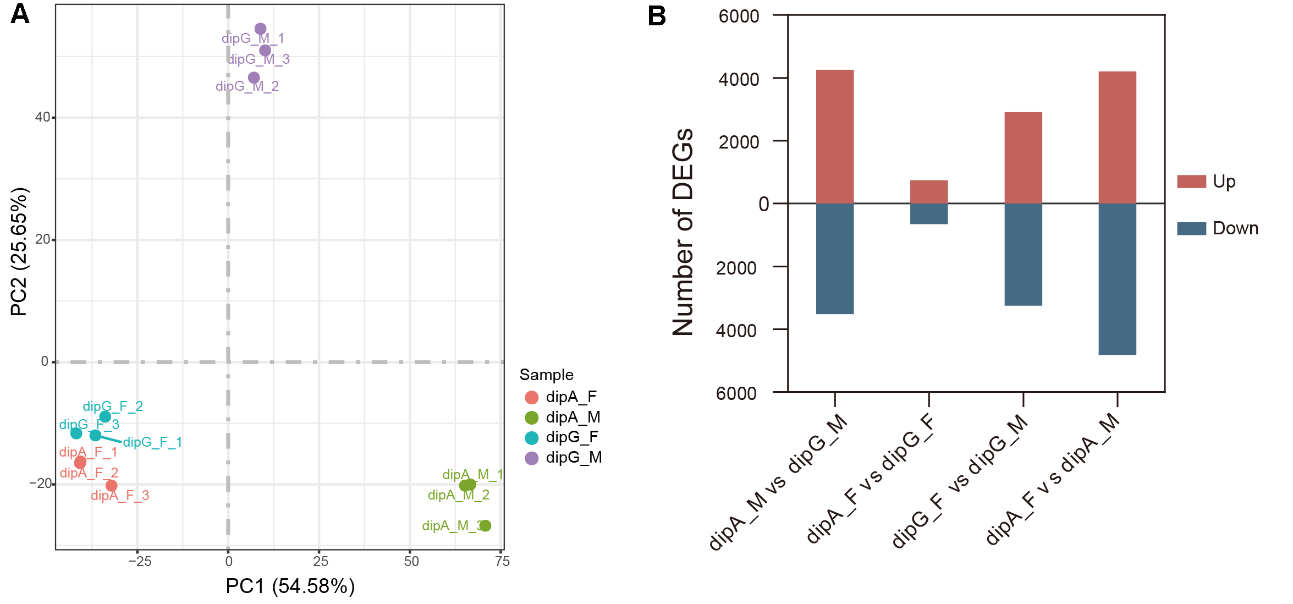


**Supplemental Figure S3.** Transcriptomic profiling of floral buds in *C. paliurus*. (A) Principal component analysis (PCA) of RNA-seq data across 12 floral samples. (B) Number of DEGs identified from different comparisons.


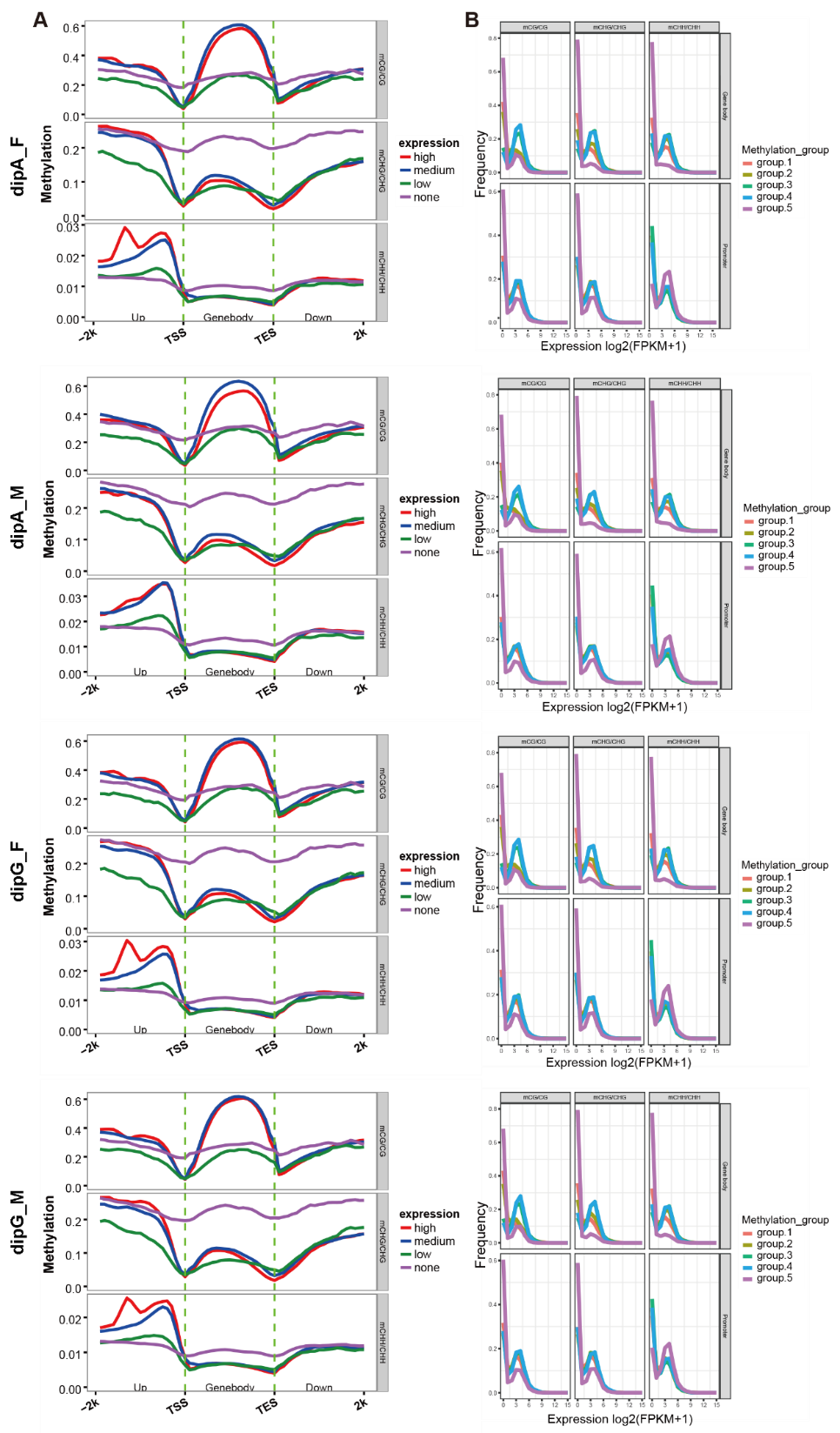


**Supplemental Figure S4.** Relationship between DNA methylation and gene expression in *C. paliurus*. (A) Distributions of methylation levels in three contexts within the gene body, upstream, and downstream regions based on four expression levels: none (FPKM < 1), low (1 < FPKM < low quartile), medium (low quartile < FPKM < upper quartile), and high (FPKM > upper quartile). TSS, the transcription start sites; TES, the transcription end sites. (B), Expression profiles of methylated and unmethylated genes. Methylation genes were divided into five groups: group 1 (< low quintile), group 2 (low quintile to second quintile), group 3 (the third quintile to fourth quintile), group 4 (the fourth quintile to upper quintile), and group 5 (> upper quintile).





**Supplemental Figure S5.** Correlation analysis between DNA methylation and the expression levels of DNA methyltransferase and demethylase genes.


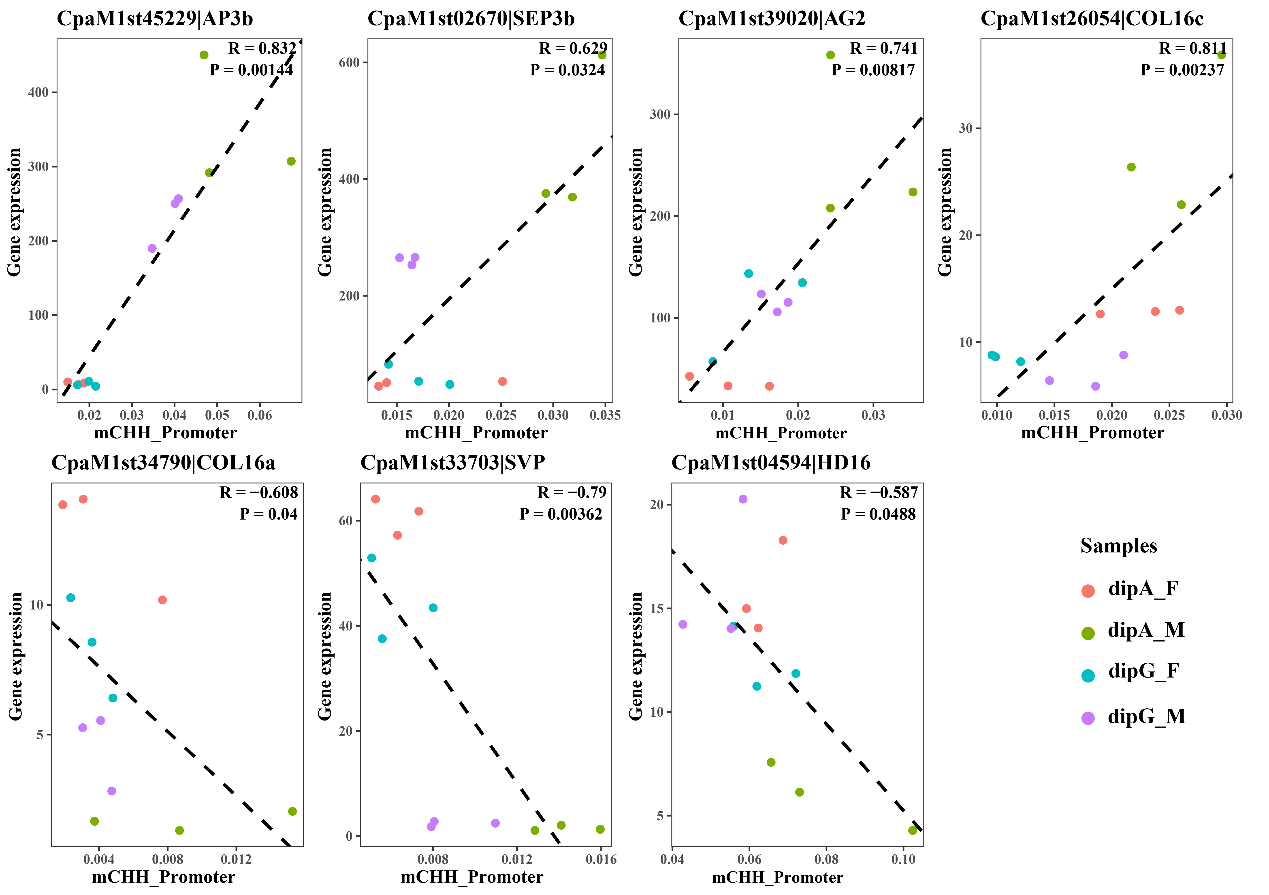


**Supplemental Figure S6.** Correlation analysis between the CHH methylation levels and expression levels in flowering pathway genes.


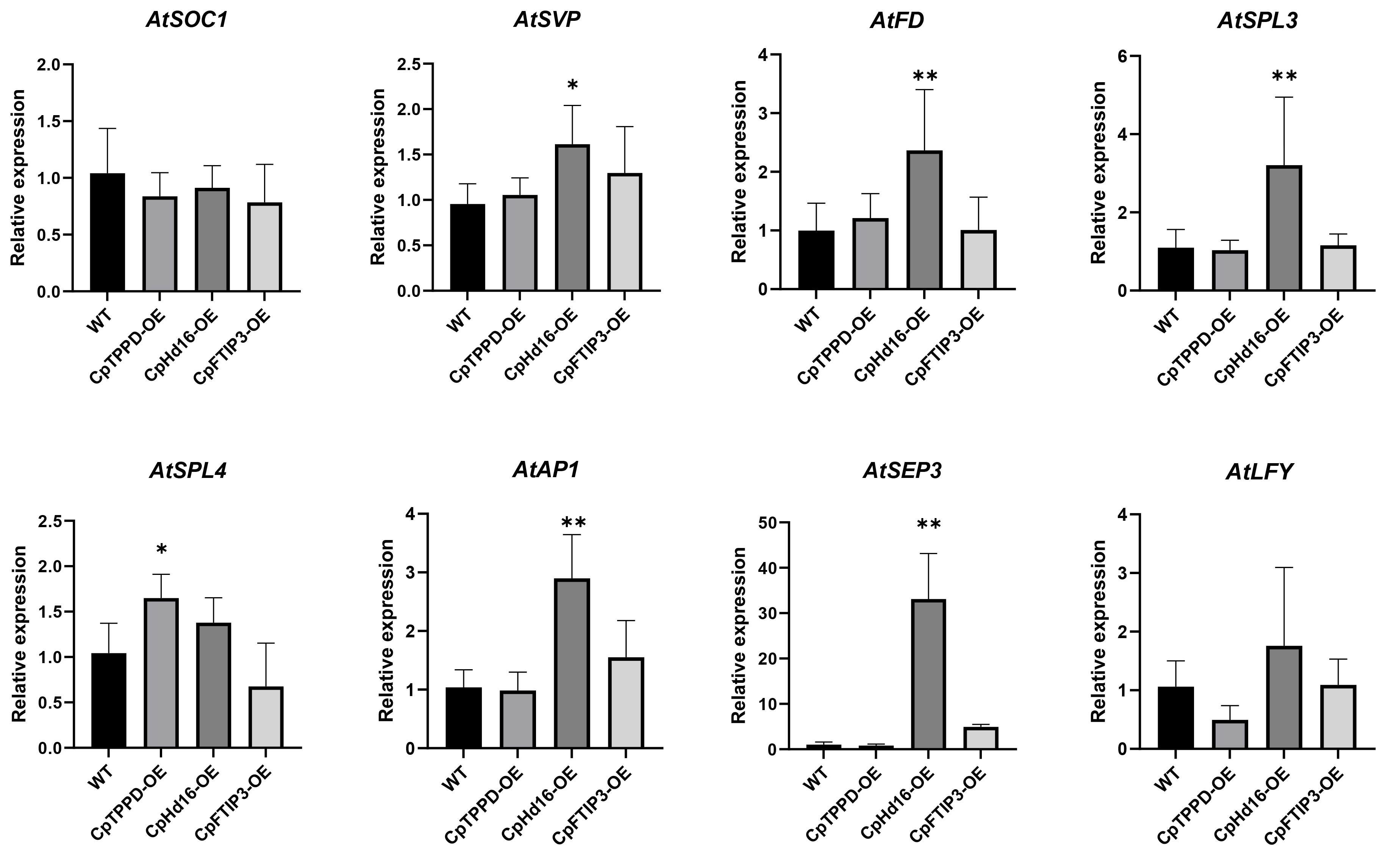


**Supplemental Figure S7.** Relative expression levels of flowering-related genes in wild-type (WT) and transgenic *Arabidopsis*. Data are mean ± SD, Wilcoxon test: **P* < 0.05, ***P* < 0.01 vs. WT.


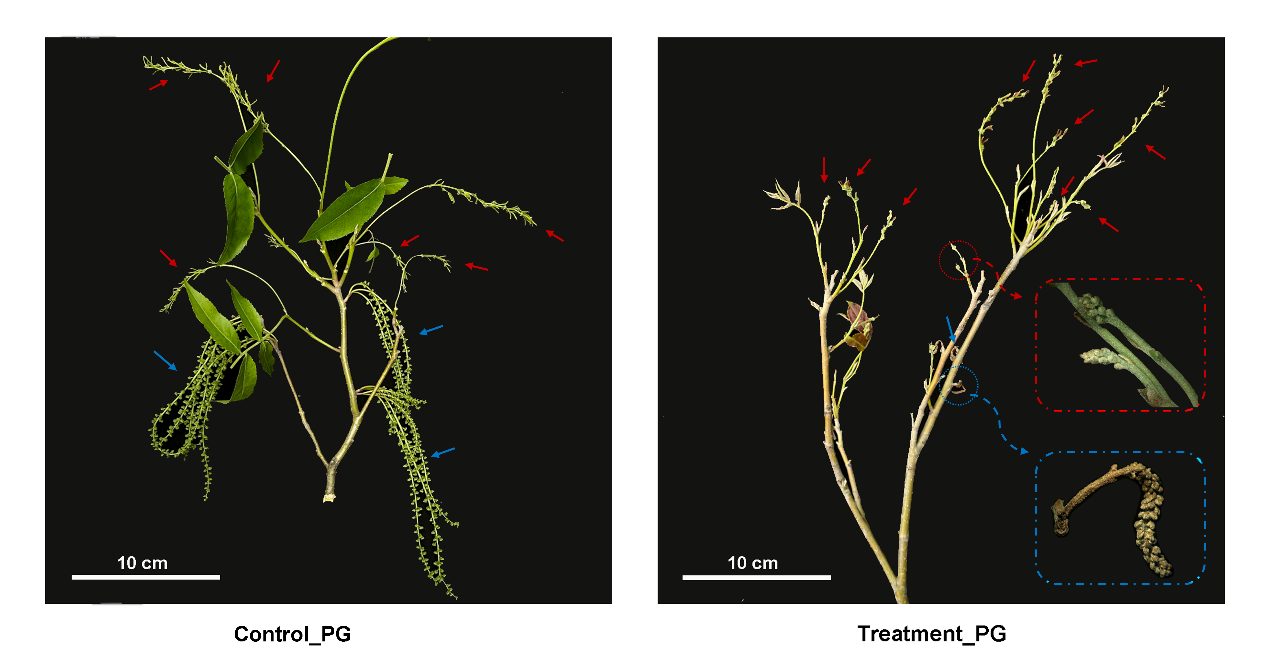


**Supplemental Figure S8.** The effect of 5-azaC treatment on the flowering of protogyny *C. paliurus*. Scale bar, 10cm; red arrows, indicate the female flowers; blue arrows, represent the male flowers.


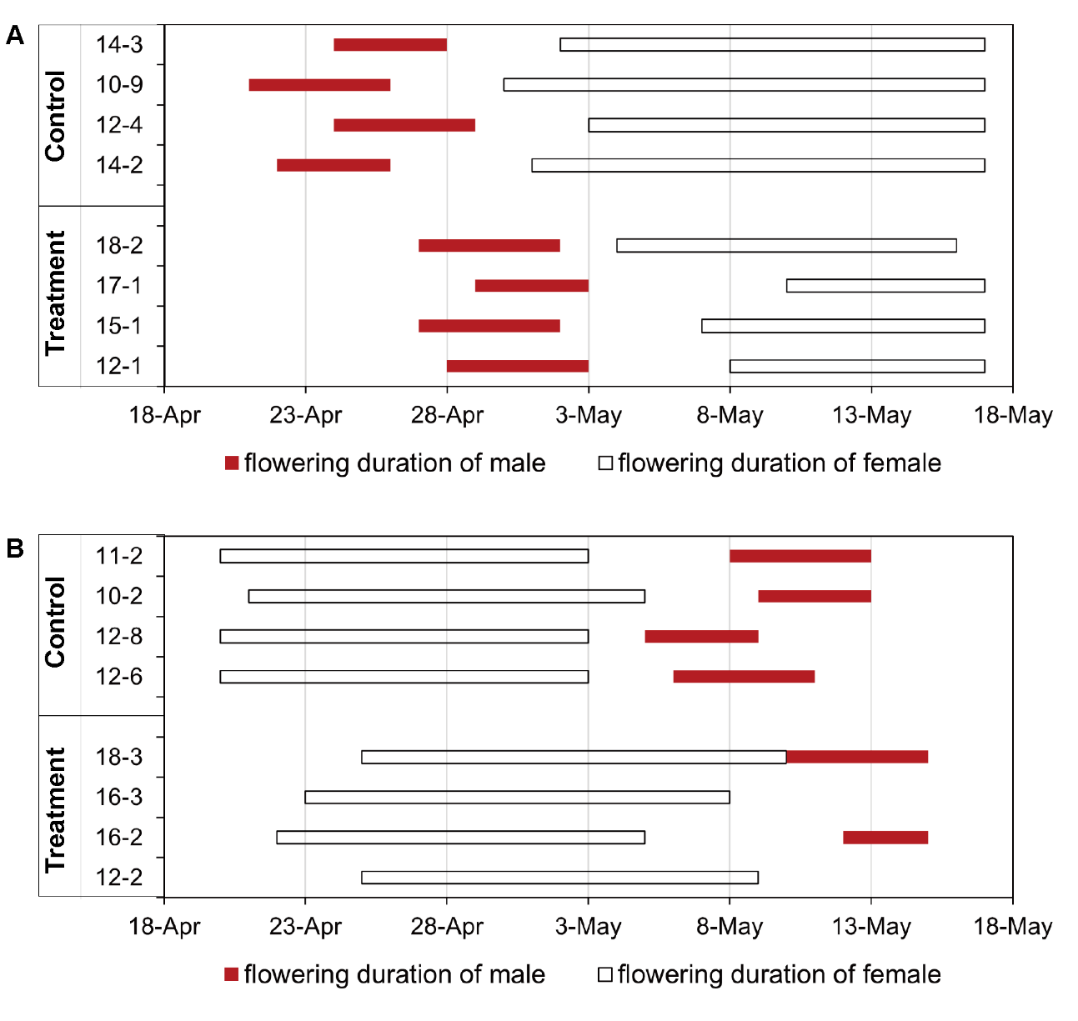


**Supplemental Figure S9.** Phenological changes in the flowering of male and female flowers of *C. paliurus* after 5-azaC treatment in protandry (A) and protogyny (B).


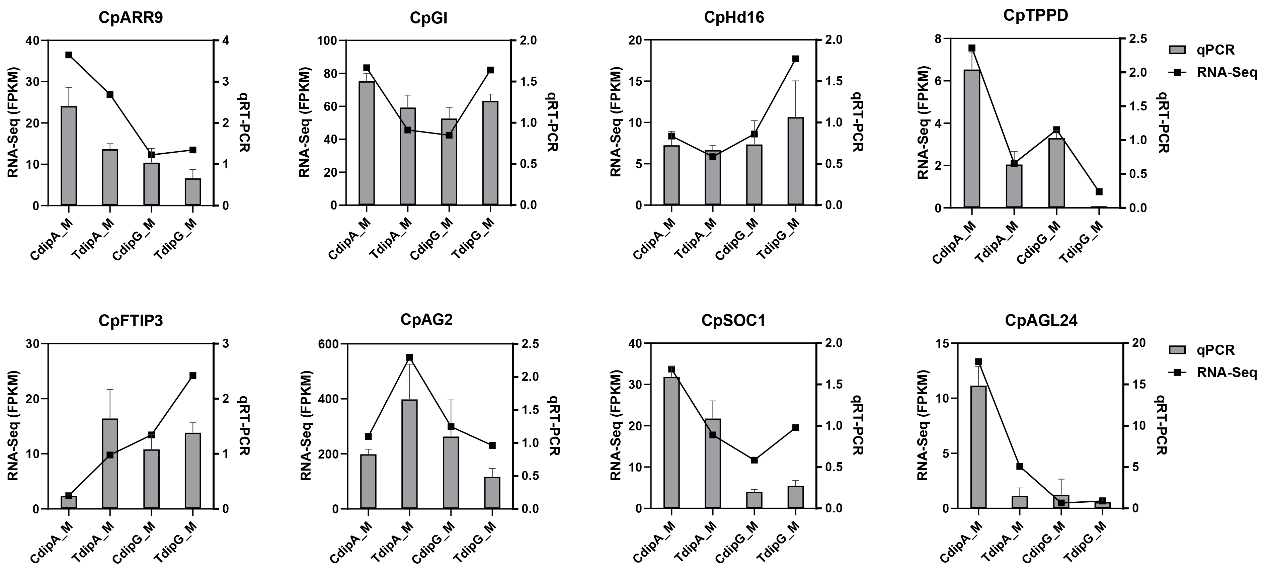


**Supplemental Figure S10.** Expression levels of flowering-related genes detected by RNA-seq and RT-qPCR.


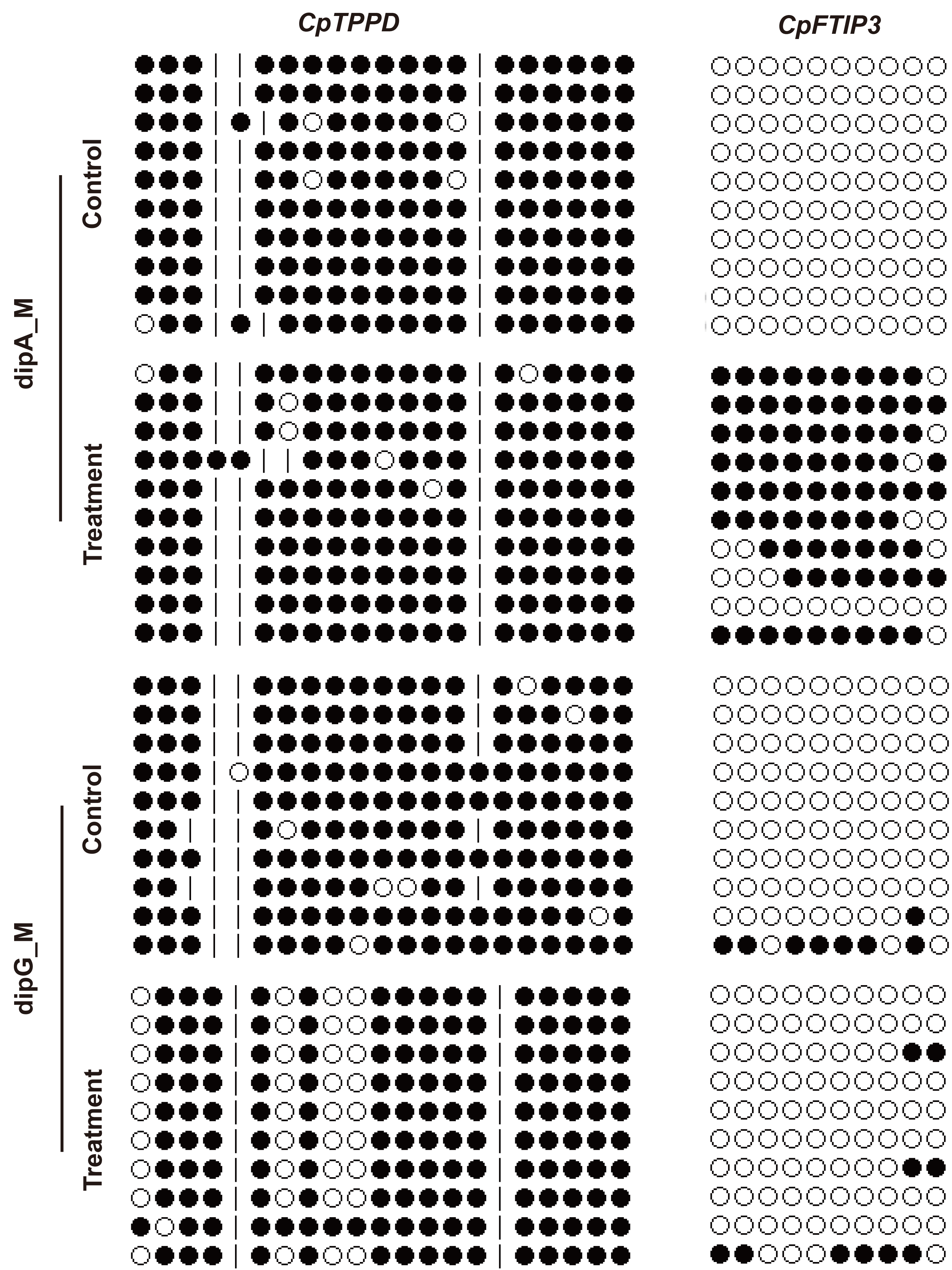


**Supplemental Figure S11.** DNA methylation patterns of CpG island in the *CpTPPD* promoter and *CpFTIP* gene body regions. Bisulfite sequencing PCR results from dipA_M and dipG_M samples. Each row represents an individual cloned sequence; each column corresponds to a single CpG site within the CpG islands. Open and filled circles indicate unmethylated and methylated cytosines, respectively.


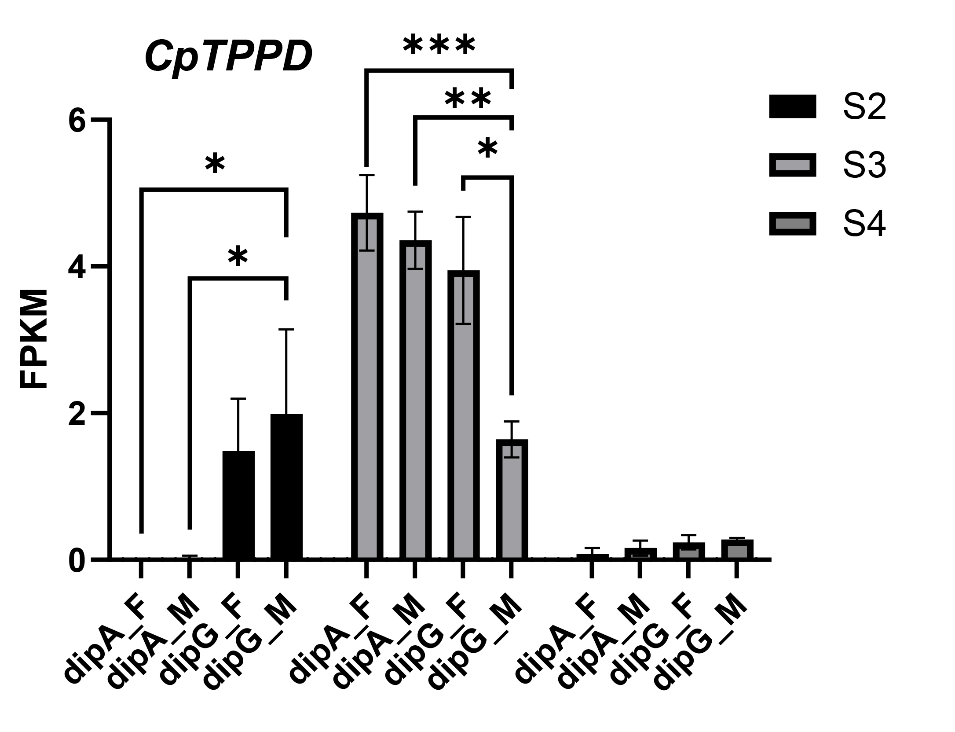


**Supplemental Figure S12.** Expression levels of *CpTPPD* in female and male flowers of *C. paliurus* across developmental stages. S1, bud break stage; S2, inflorescence elongation stage; S3, mature stage.

| **Supplemental Table S1** Summary of bisulfate sequencing and reads alignment | | | | | | | | | |
| --- | --- | --- | --- | --- | --- | --- | --- | --- | --- |
| **Sample name** | **Raw reads** | **Row bases (G)** | **Clean reads** | **Clean ratio (%)** | **BS Conversion rate (%)** | **Mapped reads** | **Mapping rate (%)** | **Unique mapping rate (%)** | **Duplication rate (%)** |
| dipA_F_1 | 70234866 | 21.07 | 68138013 | 88.42 | 99.645 | 51908469 | 76.18 | 67.13 | 9.06 |
| dipA_F_2 | 61761024 | 18.53 | 59895293 | 88.45 | 99.646 | 45666955 | 76.24 | 66.95 | 9.29 |
| dipA_F_3 | 81915177 | 24.57 | 78751638 | 87.3 | 99.645 | 58309287 | 74.04 | 65.47 | 8.57 |
| dipA_M_1 | 67171997 | 20.15 | 64858879 | 87.94 | 99.486 | 48442812 | 74.69 | 66.33 | 8.36 |
| dipA_M_2 | 78550286 | 23.57 | 76511164 | 88.97 | 99.649 | 58589654 | 76.58 | 68.64 | 7.93 |
| dipA_M_3 | 65857894 | 19.76 | 63814594 | 88.31 | 99.666 | 48216642 | 75.56 | 66.82 | 8.74 |
| dipG_F_1 | 66098319 | 19.83 | 63885876 | 87.9 | 99.597 | 47877636 | 74.94 | 66.53 | 8.41 |
| dipG_F_2 | 64716297 | 19.41 | 62769228 | 88.3 | 99.657 | 47094845 | 75.03 | 67.03 | 8 |
| dipG_F_3 | 65700256 | 19.71 | 63495859 | 88.03 | 99.699 | 47994201 | 75.59 | 66.83 | 8.76 |
| dipG_M_1 | 66636670 | 19.99 | 64652654 | 88.54 | 99.628 | 49258144 | 76.19 | 68.15 | 8.04 |
| dipG_M_2 | 63437118 | 19.03 | 61400906 | 88.23 | 99.657 | 46445427 | 75.64 | 67.55 | 8.1 |
| dipG_M_3 | 64838719 | 19.45 | 62969752 | 88.64 | 99.663 | 48183560 | 76.52 | 68.2 | 8.32 |

| Supplemental Table S2. Primers used in this study | | |
| --- | --- | --- |
| **Primer name** | **Primer sequence (5′→3′)** | **Utility** |
| *CpFTIP3* | F: gacagcccagatcaactagtATGGAGGAGATAAGGCTGCA | Amplification |
|  | R: caccatggatcccccgggTCGACCAGGATGGGCACC |  |
| *CpHd16* | F: gacagcccagatcaactagtATCTTCGGAGACCTTGTGTTG |  |
|  | R: caccatggatcccccgggTCGACCAGGATGGGCACC |  |
| *CpTPPD* | F: gacagcccagatcaactagtATGACTAAGCAGAATGTGGTGGTTT |  |
|  | R: caccatggatcccccgggGTGCATGCATCTTAAATTAATTTGG |  |
| *CpFTIP3* | F: CTTGGAGGGGGAAAGGTCAC | RT-qPCR |
|  | R: TTTGCAGGCAAATCCTTGGC |  |
| *CpHd16* | F: TGCAATAGCTGGAGCGACAA |  |
|  | R: GCTGTGCCCATTGCAGTAAC |  |
| *CpTPPD* | F: ATGGTGCTCAGTGCGTATCC |  |
|  | R: TCCCACTTGATGGTTGGACG |  |
| *Atactin-2* | F: GGTGATGGTGTGTCTCACACTG |  |
|  | R: GAGGTTTCCATCTCCTGCTCGTAG |  |
| *AtFT* | F: CTAGCAACCCTCACCTCCGA |  |
|  | R: CTGCCAAGCTGTCGAAACAA |  |
| *AtSOC1* | F: TCAATCGAGGAGCTGCAACA |  |
|  | R: AGCTAGAGCTTTCTCCTTTTGCT |  |
| *AtSVP* | F: CCGGAAAACTGTTCGAGTTCTG |  |
|  | R: TCACTGTTCTCAACCAGCTGTA |  |
| *AtAP1* | F: TCTCAACATGGGTGGTCTGT |  |
|  | R: AAATGCTTCATGCGGCGAAG |  |
| *AtLFY* | F: CGGCGAAGATAGCGGAGTTA |  |
|  | R: ACCCTGTCCAATCATCTTCTTG |  |
| *AtFD* | F: CACCTCTTTCGAGGCTCTGG |  |
|  | R: CTCGTTTGTATAAGCCTGTTTCC |  |
| *AtSPL3* | F: TGCCAGTTTCATGCCAAAGC |  |
|  | R: GCCTTCTCTCGTTGTGTCCA |  |
| *AtSPL4* | F: GTCAACAATGCAGTAGGTTTCA |  |
|  | R: ATGACAGAAGAGAGAGAGCAGAC |  |
| *AtSEP3* | F: ACGCATGCTGACTGAGACAA |  |
|  | R: TTGCCCCTGATACCCGATCT |  |
| *CpARR9* | F: AGCAACCCAGAAACACCCTC |  |
|  | R: AAGGCACATTCTCGGATGACA |  |
| *CpGI* | F: TTGACTCAGCTGTTCGCCAT |  |
|  | R: CTTGCATCGCATCAAGACCG |  |
| *CpFTIP3* | F: CAGTCTTCGGTGCTTGAGGT |  |
|  | R: CCAGTGGACTATCTGGGGGA |  |
| *CpAG2* | F: GCGGTCGCCTTTACGAGTAT |  |
|  | R: GCCTCTCGCTGGTAGAACTG |  |
| *CpAGL24* | F: CTGGACTTAGCCGTGTGCTT |  |
|  | R: CTGATTCCGACGACATGCCT |  |
| *CpHd16* | F: CAAGCTTGTGGGACGTTTGG |  |
|  | R: ACATCCCCGTGCACATATCC |  |
| *CpSOC1* | F: AGGACCTGGGTCTCGAAGAA |  |
|  | R: GCGGCCTATAGACTCTGCTG |  |
| *Cp18S* | F: AGTATGGTCGCAAGGCTGAAA |  |
|  | R: CAGACAAATCGCTCCACCAA |  |
| *CpFTIP3* | Inner F: GAGATTAATTTTTATTTTGGAGG | Bisulfite sequencing PCR |
|  | Inner R: CTTTAAAAAAAACAAACACCTAA |  |
|  | Outer F: GATGTATATTAGGTTTTTATTAGAAGAT |  |
|  | Outer R: TTATCCTTCACTATAACCTCAAAC |  |
| *CpTPPD* | Inner F: GGTATTTATTAAATAGTAATTAGGATT |  |
|  | Inner R: AACTTAAAAAATCCAAAAAAAC |  |
|  | Outer F: TTAGTATGAAAAATAGTTTTTGGT |  |
|  | Outer R: AATCAATACACAAAAAAATTATTAA |  |
